# *i*-GONAD: a robust method for *in situ* germline genome engineering using CRISPR nucleases

**DOI:** 10.1186/s13059-018-1400-x

**Published:** 2018-02-26

**Authors:** Masato Ohtsuka, Masahiro Sato, Hiromi Miura, Shuji Takabayashi, Makoto Matsuyama, Takayuki Koyano, Naomi Arifin, Shingo Nakamura, Kenta Wada, Channabasavaiah B. Gurumurthy

**Affiliations:** 1 Department of Molecular Life Science, Division of Basic Medical Science and Molecular Medicine, School of Medicine, Tokai University, Isehara, Kanagawa Japan; 2 Center for Matrix Biology and Medicine, Graduate School of Medicine, Tokai University, Isehara, Kanagawa Japan; 3 The Institute of Medical Sciences, Tokai University, Isehara, Kanagawa Japan; 4 Section of Gene Expression Regulation, Frontier Science Research Center, Kagoshima University, Kagoshima, Japan; 5 Laboratory Animal Facilities & Services, Preeminent Medical Photonics Education & Research Center, Hamamatsu University School of Medicine, Hamamatsu, Shizuoka, Japan; 6 Division of Molecular Genetics, Shigei Medical Research Institute, Minami-ku, Okayama, Japan; 7 Department of Applied Biochemistry, School of Engineering, Tokai University, Hiratsuka, Kanagawa Japan; 8 Division of Biomedical Engineering, National Defense Medical College Research Institute, Tokorozawa, Saitama, Japan; 9 Department of Bioproduction, Tokyo University of Agriculture, Abashiri, Hokkaido Japan; 10 Mouse Genome Engineering Core Facility, Vice Chancellor for Research Office, University of Nebraska Medical Center, Omaha, NE USA; 11 Developmental Neuroscience, Munroe Meyer Institute for Genetics and Rehabilitation, University of Nebraska Medical Center, Omaha, NE USA

**Keywords:** *In vivo* electroporation, CRISPR, GONAD, Knock-in, Transgenic mouse, Long ssDNA, *Easi-*CRISPR

## Abstract

We present a robust method called improved-Genome editing via Oviductal Nucleic Acids Delivery (*i*-GONAD) that delivers CRISPR ribonucleoproteins to E0.7 embryos via *in situ* electroporation. The method generates mouse models containing single-base changes, kilobase-sized deletions, and knock-ins. The efficiency of *i*-GONAD is comparable to that of traditional microinjection methods, which rely on *ex vivo* handling of zygotes and require recipient animals for embryo transfer. In contrast, *i-*GONAD avoids these technically difficult steps, and it can be performed at any laboratory with simple equipment and technical expertise. Further, *i*-GONAD-treated females retain reproductive function, suggesting future use of the method for germline gene therapy.

**Electronic supplementary material:**

The online version of this article (10.1186/s13059-018-1400-x) contains supplementary material, which is available to authorized users.

## Background

Recent advances in genome editing using clustered regularly interspaced short palindromic repeats (CRISPR)/CRISPR associated protein 9 (Cas9) enable production of gene knock-out animals easily and rapidly [1–3]. CRISPR animal genome engineering methods include three broad steps: mating of super-ovulated females and isolation of zygotes, microinjection of genome editing components into the zygotes, and transfer of microinjected zygotes into the oviducts of pseudopregnant females [1, 2]. These steps require (1) a very high level of technical expertise by the technicians who perform these procedures and (2) expensive apparatus, including micromanipulators. Because of the complex nature of the protocol, animal genome engineering experiments are difficult to perform in individual laboratories, and are typically performed in centralized cores, where highly trained personnel offer genome engineering services on a day-to-day basis. The development of methods that circumvent such complex steps enables animal genome engineering technologies to be performed by many more laboratories, not just cores. Some groups have investigated the use of *in vitro* electroporation of zygotes as an alternative to microinjection, and they successfully produced genome-edited fetuses and pups using this approach [4–9]. Electroporation of zygotes overcomes the microinjection step, but this strategy still requires the other two difficult steps: isolation of zygotes for *ex vivo* handling and their transfer back into pseudopregnant females. We recently demonstrated that all three steps can be bypassed by performing *in situ* electroporation of zygotes.

To simplify germline genome editing, we developed a method called Genome-editing via Oviductal Nucleic Acids Delivery (GONAD), which does not require isolation of zygotes or their *ex vivo* handling for microinjection and subsequent transfer to recipient females [10]. GONAD is performed on pregnant mouse females bearing E1.5 (2-cell stage) embryos. The ovaries and oviducts are surgically exposed through an incision at a dorsolateral position, and genome editing reagents are injected into the oviductal lumen using a glass capillary pipette. Immediately after solution injection, the entire oviduct is subjected to electroporation using tweezer-type electrodes. After electroporation, the ovaries and oviducts are returned to their original position and the incision is sutured. The *in situ* genome-edited embryos subsequently develop to term, and the offspring are genotyped for the targeted mutation. We demonstrated that it is possible to create *indel* mutations at target loci in some of the fetuses with 28% efficiency (7/25) [10]. When developing this strategy, we realized that the method could be significantly improved by systematically testing various parameters, enabling the method to achieve precise genome editing. The improvements that needed to be achieved included methods for (1) small point mutation knock-in and large cassette knock-ins (not just *indels*); (2) germline transmission of the founder (G0) mutations; (3) reduction of mosaicism, which typically occurs if genome editing happens at the 2-cell stage and beyond; (4) testing of additional commercially available electroporators (the model used in our initial studies is no longer available; (5) ascertainment of the fertility of females following the GONAD procedure; and (6) determining whether the GONAD method works with AsCpf1, the second most commonly used CRISPR family nuclease.

In this study, we made major modifications to improve GONAD. We termed the new method *i*mproved GONAD (*i*-GONAD), because it offers much higher genome editing efficiencies. We demonstrate that the *i*-GONAD approach can be used to create germline-modified G1 offspring with genetic changes including large deletions and knock-ins. Furthermore, we demonstrate that *i*-GONAD is robust because many commonly used electroporators can be used. These features make *i*-GONAD easily adaptable for all laboratory personnel, including beginners or students who do not possess the skills needed to operate specialized equipment such as micromanipulators.

## Results

### GONAD on day 0.7

**Fig. 1:**
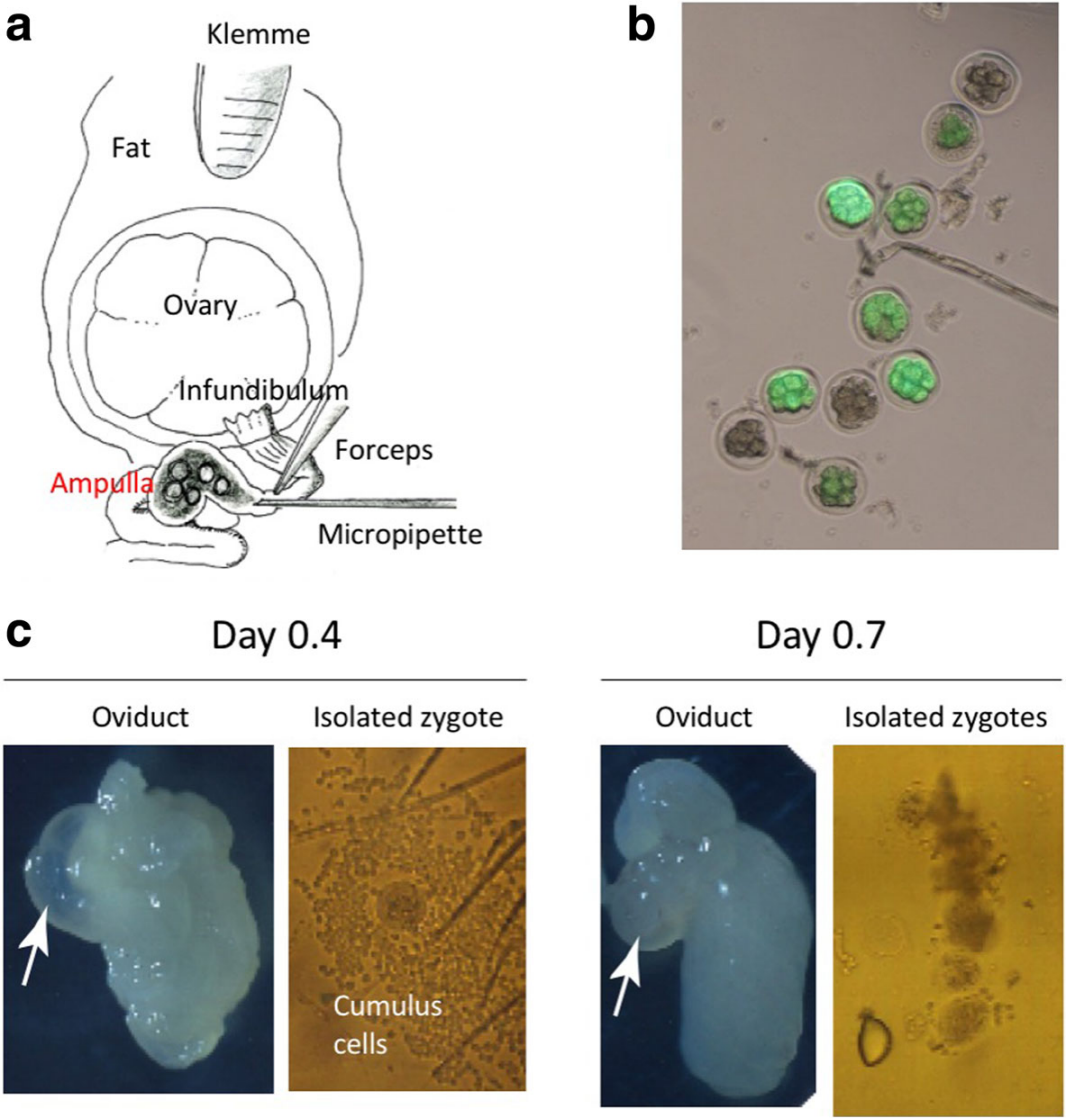
Evaluation of earlier time points for performing GONAD. **a** Diagrammatic illustration showing the anatomical structures of ovary and oviduct and the surgical equipment used for GONAD procedure. A small amount of solution is injected by direct insertion of a glass micropipette through oviduct wall located at the region between the ampulla and infundibulum. Immediately after injection, *in vivo* electroporation is performed on the entire oviduct. **b** Detection of eGFP fluorescence in 8-cell to morula embryos after delivery of eGFP mRNA via GONAD procedure. The eGFP fluorescence in preimplantation embryos, isolated 2 days post GONAD procedure performed on naturally mated Jcl:MCH(ICR) female at 0.7 day of pregnancy. **c** Oviducts and zygotes dissected on days 0.4 (*left panel*) and 0.7 (*right panel*). Note that the oviduct dissected on day 0.4 exhibits swelling of the ampulla (*arrow*). The zygotes isolated from the day 0.4 ampulla are usually surrounded by thick layer of cumulus cells. These cells may hamper efficient uptake of exogenous nucleic acids/proteins injected intra-oviductally and subsequently electroporated. The oviduct dissected on day 0.7 exhibits shrinkage of the ampulla (*arrow*), and zygotes isolated from the day 0.7 ampulla have fewer cumulus cells, which will less likely hamper the uptake of exogenous nucleic acids/proteins upon electroporation

In our first report on the GONAD method, experiments were performed at ~ 1.5 to 1.7 day post-mating. At this stage of pregnancy, embryos are at the 2-cell stage. Delivery of genome editing reagents at this stage results in a high frequency of mosaic embryos or fetuses [10]. The ideal time to deliver gene editing components would be one that corresponds to the 1-cell stage because it would reduce genetic mosaicism. To investigate the earliest time of editing component delivery, we tested GONAD at two separate time points, day 0.4 and day 0.7. We injected 1.0–1.5 μl of a solution containing enhanced green fluorescent protein (eGFP) messenger RNA (mRNA) (1 μg/μl) and trypan blue into oviduct lumens (schematic shown in Fig. 1a) and then performed *in vivo* electroporation using a BTX T820 instrument under previously described conditions [10]. Two days after mRNA delivery, 8-cell stage embryos were isolated from the treated females and observed for the presence of eGFP fluorescence. We did not observe appreciable eGFP in any of the embryos treated at the 0.4 day time point, whereas uniform eGFP fluorescence was observed in 13 of 31 embryos treated at the 0.7 day time point (Fig. 1b). One of the reasons for the lack of success at day 0.4 could be that cumulus cells tightly surround the zygotes at this stage (Fig. 1c, left), and this may hamper efficient electrophoretic delivery of nucleic acids to zygotes. In contrast, most zygotes at day 0.7 should be devoid of thick cumulus cells (Fig. 1c, right), permitting access of the injection mix to the zygotes. These results suggested that 0.7 day (~ 16 h) post-mating would be the best time to perform GONAD.

### Generation of *Foxe3* knock-out lines by a modified GONAD protocol

**Fig. 2:**
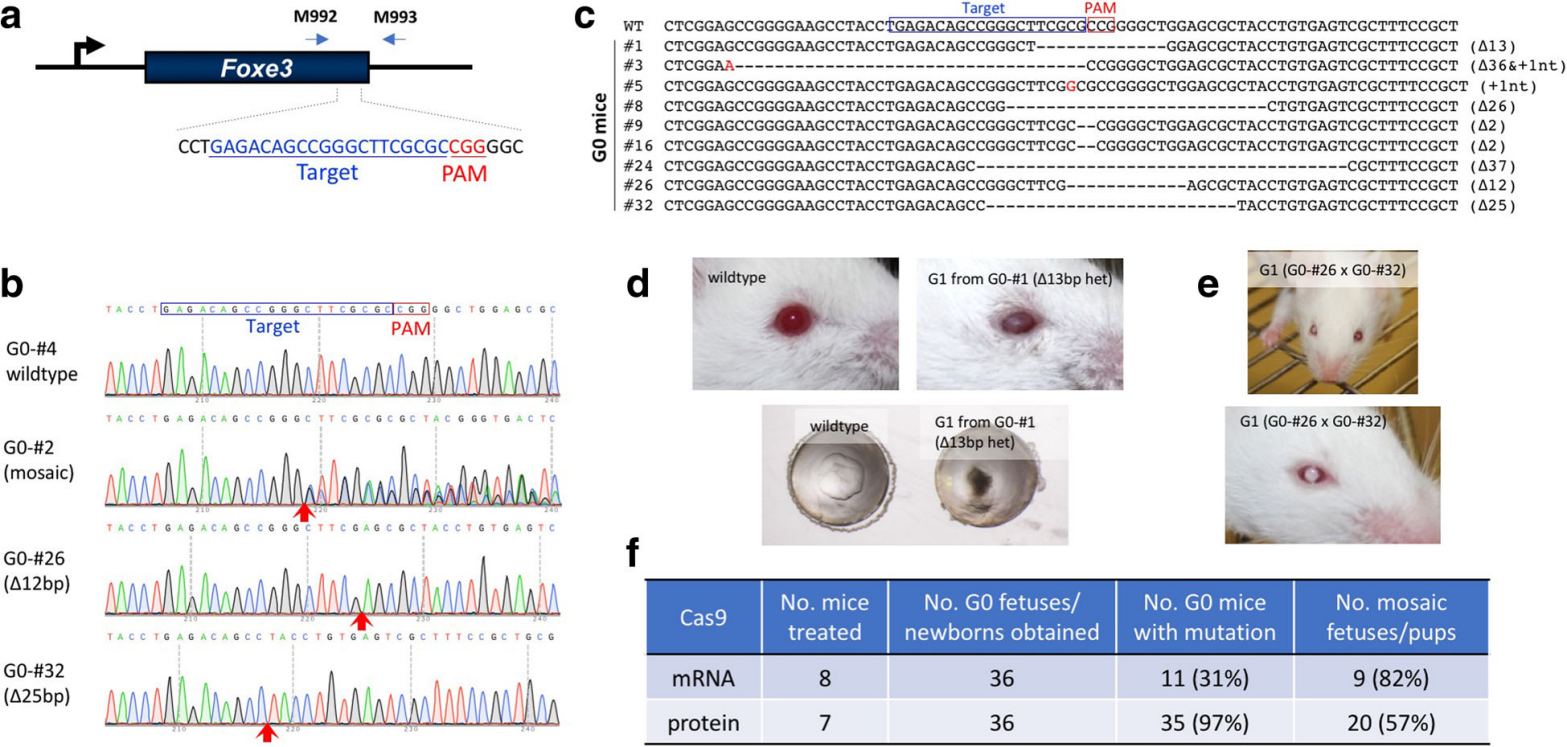
Creating gene-inactivated animal models using the GONAD method. **a** Schematic of the targeting strategy to inactivate *Foxe3* gene and the primer set used for genotyping. **b** Direct sequencing results of polymerase chain reaction (PCR) products amplified from the founder (G0) mice with the primer set shown in **a**. The *red arrows* below the electropherogram show the region with *indel* mutations. **c** Mutated *Foxe3* alleles in the G0 mice. The changes in the nucleotide sequence are shown in *red*, and the type of changes (insertions +Xnt, or deletions Δ) is indicated on the *right side* of the sequences. **d** and **e** Cataract phenotypes in the G1 mice. **f** Efficiencies of *Foxe3* gene editing. CRISPR components used were either Cas9 mRNA/sgRNA or Cas9 protein/CRISPR RNA (crRNA)/trans-activating crRNA (tracrRNA) (see Additional file 1: Table S1 for details)

Next, we asked whether the GONAD method could be used to create gene-disrupted animal models. We chose the *Foxe3* locus for gene targeting (Fig. 2a) because its inactivation causes abnormal development of the eye and cataracts in mice [11, 12]. GONAD was performed on 0.7-day pregnant Jcl:MCH(ICR) females using Cas9 mRNA and a single-guide (sg)RNA targeting the *Foxe3* gene. The electroporation conditions used were 8 pulses of 50 V at 5 ms wave length. Seven of the eight GONAD-treated females delivered pups. Sequencing of genomic DNA isolated from the ears of G0 offspring demonstrated that 11 of 36 G0 pups (31%) had a mutated allele in the target locus (Fig. 2b and c; Additional file 1: Table S1). Notably, there was still a high frequency of pups with mosaic alleles (82% [9/11]), and mosaicism was absent from only two pups (18% [2/11]; G0-#26 and -#32 in Fig. 2b). Intercrossing of G0 founders resulted in G1 offspring, some of which exhibited the expected cataract phenotype (Fig. 2d and e). Sequencing of genomic DNA isolated from G1 mice with cataracts revealed germline transmission of the mutated alleles. Taken together, these results indicate that GONAD can be used to create gene-disrupted mouse models.

**Fig. 3:**
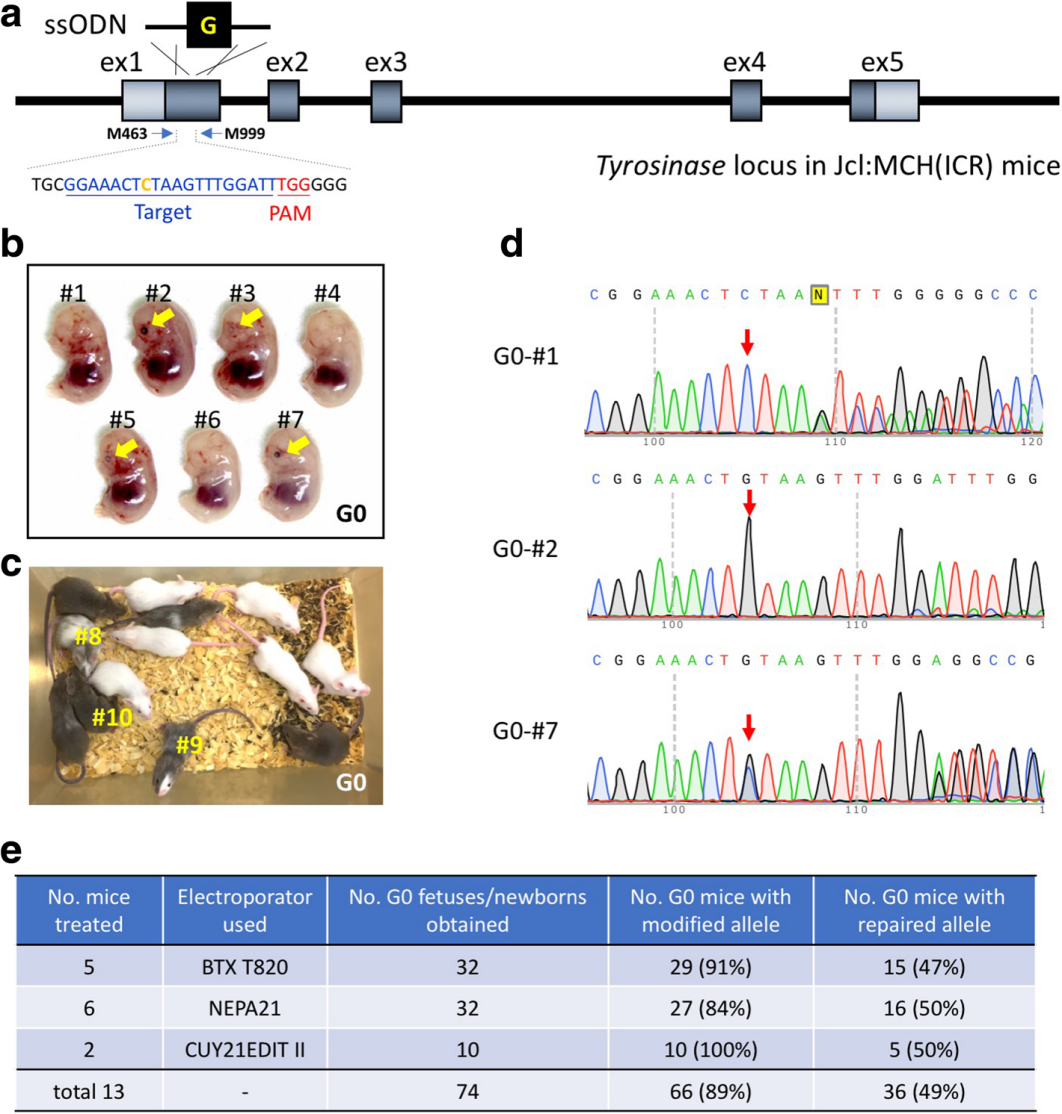
Creating small genetic change animal models using the *i*-GONAD method. Restoration of *Tyr* gene of albino Jcl:MCH(ICR) mice by single-stranded oligo donor (ssODN)-based knock-in with the *i*-GONAD method. **a** Schematic to show rescue of *Tyr* gene mutation. The target region containing the guide sequence and the genotyping primer binding sites are shown. **b** Representative E14.5 litter showing *Tyr* rescued G0 fetuses. The pigmented eyes of the fetuses are indicated by *yellow arrows*. **c** Representative *Tyr* rescued G0 mouse litters obtained from #5 female mouse in Additional file 1: Table S2. G0 mice indicated in # numbers (shown in *yellow*) were used for germline transmission analysis (see details in Additional file 1: Figure S2). **d** Direct sequencing results of PCR products amplified from the G0 fetuses in **b**. The positions for mutated/corrected nucleotides are indicated by *red arrows*. **e** Efficiencies of *Tyr* gene editing. Electroporators from three different suppliers were used (see Additional file 1: Table S2 for details)

### Higher genome editing efficiency using CRISPR RNA (crRNA) + trans-activating crRNA (tracrRNA) + Cas9 protein (ctRNP) complexes

It is now becoming increasingly clear that the use of Cas9 protein, instead of Cas9 mRNA [7, 13, 14], together with crRNA + tracrRNA (two-part guide RNA), instead of a single guide RNA (sgRNA), yields higher genome editing efficiencies [13]. We thus examined the combinatorial use of these components (crRNA + tracrRNA + Cas9 protein: ctRNP) for GONAD-mediated genome editing. We targeted the *Foxe3* gene, as an example, using the same guide sequence as in the previous experiment except that annealed crRNA + tracrRNA were used in place of sgRNA, and Cas9 protein was used in place of Cas9 mRNA. A mixture of ctRNP complexes was injected into the oviduct lumen of seven pregnant Jcl:MCH(ICR) females at day 0.7. The oviduct was electroporated *in vivo* using the same electroporator as before or using the CUY21Edit II electroporator. Embryos were isolated at E13.5 or E17.5. All seven females contained fetuses (totaling 36). Surprisingly, nearly all had *indel* mutations within the *Foxe3* target sequence (35/36, 97%), which was much higher than the frequency when Cas9 mRNA was used (31%, *p* < 0.001) (Fig. 2f; Additional file 1: Table S1). Note that 57% (20/35) of fetuses exhibited mosaicism. Although this is lower than the frequency obtained when using Cas9 mRNA (82%), it was not statistically significant (*p* = 0.139). These results suggest that combinational use of crRNA, tracrRNAs, and Cas9 protein (ctRNP) offers the highest efficiency of genome editing with GONAD. We termed this RNP-based GONAD as *i*mproved GONAD (*i*-GONAD).

### Generation of gene-corrected animal models using *i*-GONAD

Next, we asked whether *i*-GONAD could be used to make small genetic changes. We chose the codon 103 of the *Tyrosinase* (*Tyr*) gene as an example. The mice containing codon T**G**T (cysteine) at this location will have normal pigmentation in coat and eyes (regarded as the wild-type phenotype, for example, C3H/He strain mice), and mice with T**C**T (serine) will have the non-pigmented phenotype of albino coat color and clear eyes (regarded as a mutant phenotype, for example, Jcl:MCH(ICR) strain) [15], due to reduced tyrosinase enzyme activity. We designed a gRNA for a region spanning the point mutation and constructed a single-stranded oligo donor (ssODN) that corresponds to the wild-type sequence of *Tyr* (Fig. 3a) to rescue the mutant phenotype in Jcl:MCH(ICR) strain (non-pigmented) to the wild-type (pigmented) phenotype.

*i*-GONAD was performed in five pregnant Jcl:MCH(ICR) females. A total of 32 offspring from these females were harvested at different stages of gestation (from E14.5 to E19.5), or postnatally. Fifteen (47%) of these samples exhibited the expected phenotype of dark eye pigmentation (in fetuses) or agouti coat color (in newborn pups; Fig. 3b and c). Sequence analysis demonstrated that at least one allele had the corrected sequence (C to G) at the target site in all of the offspring with a normal pigmentation phenotype (Fig. 3d; Additional file 1: Table S2).

The original experiments were performed using a BTX T820 electroporator, a model that is no longer manufactured. We therefore tested two newer electroporators, NEPA21 (Nepa Gene Co) and CUY21EDIT II (BEX Co). Six females were subjected to *i*-GONAD using NEPA21. This yielded 32 offspring, of which 16 (50%) showed the expected genetic change and the phenotypic change of eye pigmentation or coat color (Fig. 3e; Additional file 1: Table S2). Two females were subjected to *i*-GONAD using the CUY21EDIT II electroporator, and the fetuses were analyzed at E14.5. Of 10 fetuses obtained, 5 (50%) showed the expected genetic change as well as the phenotypic change (eye pigmentation or coat color) (Fig. 3e; Additional file 1: Table S2). Highly consistent genome editing efficiencies (47%, 50%, and 50%) were thus obtained from three different electroporators (Additional file 1: Figure S1), demonstrating the robustness and reproducibility of *i*-GONAD. These results suggest that *i*-GONAD can be used for high-efficiency gene correction via co-delivery of a ssODN repair donor.

Eighty-three percent (30/36) of the offspring with the pigmentation phenotype were found in three experiments to have *indel* mutations around the target region of their second allele (Fig. 3d). Of note, 79% (30/38) of the unpigmented offspring thought not to be repaired had *indel* mutations. Considering gene correction and *indels* together, a total of 89% (66/74) of G0 offspring were genome-edited (Fig. 3e; Additional file 1: Table S2). These results further support the conclusion that *i*-GONAD yields very high efficiencies of genome editing.

Three representative founder mice containing the repaired allele (G0-#8: 5% mosaic, G0-#9: 60% mosaic, G0-#10: 100% mosaic [based on the coat color]; Fig. 3c) were bred with Jcl:MCH(ICR) mice to assess germline transmission of the repaired allele. Although we did not obtain rescued progeny from G0-#8 (0/43), pups exhibiting an agouti coat color were obtained from founders G0-#9 and -#10 (#9 [8/18] and #10 [30/30]) (Additional file 1: Figure S2).

### Comparison of the genome editing efficiencies between *i-*GONAD and microinjection methods

We compared the genome editing efficiency of *i-*GONAD with that of the microinjection-based approach. For this study, we used the *i-*GONAD dataset presented above and compared it with microinjection of isolated zygotes. Oocytes from 35 super-ovulated Jcl:MCH(ICR) females were *in vitro* fertilized (IVF) to generate zygotes for microinjection. We injected 339 such zygotes with CRISPR reagents and cultured them. Of these 242 (71%) advanced to the 2-cell stage, and they were then transferred to 12 recipient females (Additional file 1: Table S3). The females were euthanized at E14.5 or E15.5. Of 62 fetuses recovered, 32 (52%) had pigmented eyes. Sequencing analysis showed that the fetuses with pigmented eyes had the corrected sequence at the target site. Considering gene correction and *indels* together, a total of 79% (49/62) of G0 fetuses were genome-edited. These data, directly comparing genome editing using *i*-GONAD with standard microinjection-based techniques, clearly demonstrate that the efficiency is comparable between the two strategies (*p* = 0.103). There was a somewhat lower mosaicism in genome-edited fetuses from microinjection (21/49, 43%; Additional file 1: Table S3) compared with the *i*-GONAD approach (40/66, 61%; Additional file 1: Table S2), but the difference was not statistically significant (*p* = 0.059). Notably, the standard microinjection-based approach needed about 2.5 times more animals when the Jcl:MCH(ICR) strain was used: 20 mice were required to obtain 10 correctly genome-edited mice (11 females as egg donors + 1 male as sperm donor for IVF + 4 pseudopregnant females + 4 vasectomized males), whereas *i*-GONAD used only 8 mice (4 embryo donors mated with 4 stud males).

### Generation of large genomic deletion animal models using *i*-GONAD

**Fig. 4:**
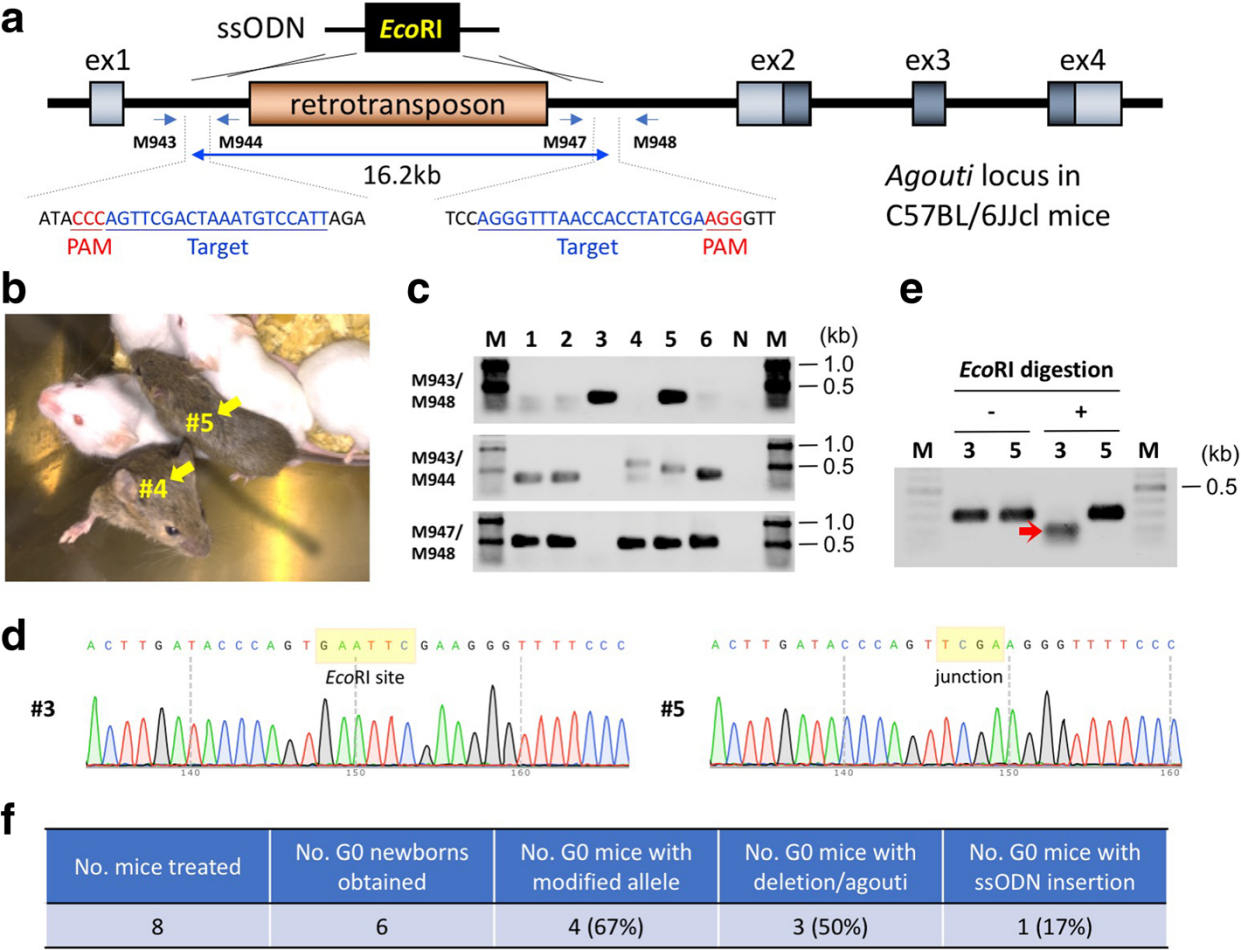
Creating large deletion using the *i*-GONAD method. **a** Schematic diagram showing deletion of 16.2-kb sequence consisting of retrotransposon in the C57BL/6JJcl mouse genome, to restore agouti phenotype. The target sequences and genotyping primers are shown. ssODN containing *Eco*RI site at the middle of the sequence was used. **b** Representative mice showing rescued agouti phenotype (indicated by *yellow arrows*). These mice were recovered through caesarean section and nursed by Jcl:MCH(ICR) foster mother with her own pups. **c** Genotyping analyses. Expected fragment sizes: M943/M948 = 290 or 295 bp (ssODN knock-in), M943/M944 = 337 bp, M947/M948 = 477 bp. **d** Direct sequencing results of PCR products amplified from the G0 mice. The position of junctional sequences is indicated by *yellow rectangles*. **e** *Eco*RI digestion of PCR products amplified from G0 mice (G0-#3 and -#5) with the M943/M948 primer set. *Red arrow* indicates digested fragment. **f** Efficiencies of *agouti* gene editing (see Additional file 1: Table S4 for details)

We next asked whether the *i*-GONAD method could be used to generate mice with large genomic deletions by targeting a retrotransposon sequence present in the first intron of the *agouti* locus in C57BL/6JJcl mice [16]. A genomic region spanning 16.2 kb containing the retrotransposon sequence was targeted for deletion by making two cleavages that flank the retrotransposon insertion site (Fig. 4a). The deletion of this region should result in a coat color change from black to agouti mice (Fig. 4b). To aid in the precise joining of the genomic ends, we provided a ssODN donor containing short homology sequences to the two cleaved ends. The ssODN also included an *Eco*RI site for use in a restriction fragment length polymorphism (RFLP) assay. We injected a solution containing Cas9 protein, two crRNA/tracrRNAs, and the ssODN into the oviduct lumen of eight E0.7 pregnant C57BL/6JJcl females and performed *in vivo* electroporation. Of these, only four females remained pregnant, from which six G0 pups were recovered through caesarean section. Genotyping of the pups revealed that three (50%) exhibited large deletions in their *agouti* locus (G0-#3 and -#5) and/or agouti coat color (G0-#4 and -#5) (Fig. 4b–d; Additional file 1: Table S4). Notably, one pup (G0-#3; 1/6 [17%]) had a correctly inserted ssODN at the target site, while another (G0-#5) showing agouti coat color did not have the insertion (Fig. 4d and e). The polymerase chain reaction (PCR) did not yield an amplicon from the G0-#4 agouti pup, suggesting that one or both of the primer binding sites may have been deleted. Interestingly, the G0-#6 pup, one that showed neither a large deletion at the target locus nor agouti coat color, had *indel* mutations at both of the cleavage sites (Fig. 4f; Additional file 1: Table S4). The percentage of individual pups that were genome-edited (large deletions and short *indels* taken together) was 67% (4/6) (Fig. 4f). These data suggest that *i*-GONAD can create large genomic deletions.

### Knocking-in long ssDNA donors using *i*-GONAD

We previously demonstrated that knocking-in long DNA sequences can be achieved efficiently by using ssDNA donors [17–19]. We asked whether long ssDNA donors could be used with *i*-GONAD to create knock-in alleles. The *Pitx3* and *Tis21* genes were selected for these knock-in experiments in order to create reporter models containing T2A-mCitrine fusion cassettes.

**Fig. 5:**
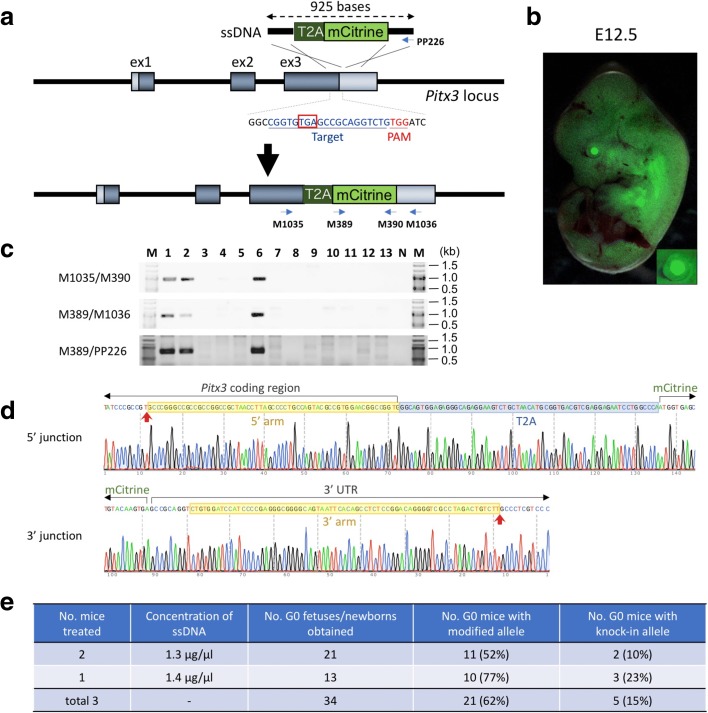
Generation of reporter knock-in mice using the *i*-GONAD method. **a** Schematic diagram showing insertion of T2A-mCitrine cassette into *Pitx3* locus. The target sequence and the genotyping primer sets are shown. A 925-base-long ssDNA synthesized by *iv*TRT method was used as the donor DNA. **b** mCitrine fluorescence in fetus collected at E12.5. The eye of the fetus is enlarged as an *inset*. **c** Example of genotyping analysis of knock-in G0 fetuses. Expected fragment sizes: M1035/M390 = 948 bp, M389/M1036 = 956 bp, M389/PP226 = 809 bp. *N* negative control, *M* size marker. **d** Representative sequencing chromatogram showing 5′ and 3′ junctional regions of the inserted cassette. The junctional sequences showing insertion derived from G0-#1 in **c** are shown. *Red arrows* indicate junctions between the arms and the genomic sequences. **e** Genome editing efficiency of the *Pitx3* locus by the *i*-GONAD method

We inserted a 783-bp T2A-mCitrine cassette immediately upstream of the stop codon of the *Pitx3* gene (Fig. 5a). The ssDNA donor was prepared using an *in vitro* transcription and reverse transcription (*iv*TRT) method described previously [17]. The concentration of Cas9 protein (1 mg/ml) and crRNA/tracrRNA (30 μM) was the same as used in the previous experiment. The ssDNA donor was used at concentrations of 1.3 or 1.4 μg/μl in the *i*-GONAD procedure. G0 fetuses were dissected at E12.5 and were observed for fluorescence under a dissecting microscope. The lenses of some fetuses exhibited fluorescence (Fig. 5b), suggestive of correct insertion of the fusion cassette. This observation is similar to the previously created knock-in model [20]. The correct insertion of the cassette at the target site was confirmed by PCR amplification and sequencing of the target region (Fig. 5c), which revealed that 15% (5/34) of samples contained the knock-in cassettes (Fig. 5d and e). Of note, the other alleles of these five samples contained *indel* mutations. Also, 16 fetuses that did not contain the insertion of the T2A-mCitrine cassette contained *indel* mutations (Fig. 5e; Additional file 1: Table S5). Collectively, the percentage of G0 individuals that had been genome-edited (knock-in and/or *indel* mutations) was 62% (21/34) (Fig. 5e). No random insertions were detected in the fetuses that did not contain the target insertion allele.

By using the same strategy as for *Pitx3*, we designed a knock-in of T2A-mCitrine for this into the *Tis21* gene, immediately upstream of its stop codon (Additional file 1: Figure S3a). The ssDNA was used at a concentration of 0.85 μg/μl, and G0 fetuses were analyzed at E14.5. Fourteen fetuses were recovered, observed for fluorescence, and genotyped by PCR. One of the fetuses exhibited fluorescence in the developing central nervous system (Additional file 1: Figure S3b) similar to a *Tis21* knock-in model created previously [21], and this fetus had the correct knock-in sequence as analyzed by PCR and sequencing. Three other fetuses that did not show fluorescence contained partial or incomplete insertions of the knock-in cassette (Additional file 1: Figure S3c–e), and two of them contained *indel* mutations in their second alleles (Additional file 1: Figure S3e; Additional file 1: Table S5). One more fetus that did not show knock-in had an *indel* mutation. Taken together, the percentage of G0 pups that were genome-edited was 36% (5/14). Significantly, random insertions were not detected in the fetuses that did not contain the target insertion allele.

### *i*-GONAD in various mouse strains

We next assessed the performance of *i*-GONAD in some inbred and hybrid mouse strains, and at additional loci (Table 1). *i*-GONAD was performed to introduce *indel* mutations in the *Tyr* gene of the C3H/HeSlc, C57BL/6NCrSlc, DBA/2CrSlc, B6D2F1/Slc, and the hybrid of the B6D2F1/Slc and C57BL/6NCrSlc strains (Additional file 1: Figure S4). We also targeted *indels* into the *Kit* gene in the C3H/HeSlc and C57BL/6NCrSlc strains (Additional file 1: Figure S5). We inserted small genetic changes via ssODNs into *Cdkn1a* and *Cdkn2a* of the C57BL/6NCrl strain (Additional file 1: Figure S6) and *Tyr* in the BALB/cAJcl strain. Results showed that *i*-GONAD produced genome editing at all the loci tested and in all of the mice strains, except for targeting the *Tyr* locus in C57BL/6NCrSlc mice. This result may be due to the difficulty of obtaining pregnant females in our C57BL/6NCrSlc colony. The overall pregnancy rates in the inbred strain females were lower. Unlike the outbred strains, many animals of inbred strains, that were confirmed to be mated by the presence of vaginal plugs, did not contain implanted embryos. Taken together, these results indicate that *i*-GONAD can be used in many mouse strains, although its success rate is strain dependent, and further optimization may be required for certain inbred strains.

**Table 1:** *i*-GONAD in various mouse strains

Type of genome editing	Concentration of CRISPR components	Electroporator used	No. of female mice treated	Strain	Locus	No. of pregnant mice	No. G0 pups/fetuses obtained	No. of G0 pups/fetuses with modified allele (%)	No. of G0 pups/fetuses with intended allele (%)
Knock-out	Cas9 protein (1 mg/ml) *Integrated DNA Technologies* (IDT)	NEPA21	7	C3H/HeSlc (inbred)	*Tyr*	3	13	12 (92%)	12 (92%) both alleles
	Tyr-crRNA (30 μM) FASMAC								
	tracrRNA (30 μM) FASMAC								
Knock-out	Cas9 protein (1 mg/ml) IDT	NEPA21	6	C3H/HeSlc (inbred)	*Kit*	4	16	11 (69%)	7 (44%) both alleles
	Kit-crRNA (30 μM) FASMAC								4 (25%) one allele
	tracrRNA (30 μM) FASMAC								
Knock-out	Cas9 protein (1 mg/ml) IDT	NEPA21	11	C57BL/6NCrSlc (inbred)	*Tyr*	0	0	0 (0%)	0 (0%)
	Tyr-crRNA (30 μM) FASMAC								
	tracrRNA (30 μM) FASMAC								
Knock-out	Cas9 protein (1 mg/ml) IDT	NEPA21	7	C57BL/6NCrSlc (inbred)	*Kit*	2	3	3 (100%)	1 (33%) both alleles
	Kit-crRNA (30 μM) FASMAC								2 (67%) one allele
	tracrRNA (30 μM) FASMAC								
Knock-out	Cas9 protein (1 mg/ml) IDT	NEPA21	10	DBA/2CrSlc (inbred)	*Tyr*	4	6	6 (100%)	6 (100%) both alleles
	Tyr-crRNA (30 μM) FASMAC								
	tracrRNA (30 μM) FASMAC								
Knock-out	Cas9 protein (1 mg/ml) IDT	NEPA21	4	B6D2F1/Slc (hybrid)	*Tyr*	4	23	20 (87%)	19 (83%) both alleles
	Tyr-crRNA (30 μM) FASMAC								1 (4%) one allele
	tracrRNA (30 μM) FASMAC								
Knock-out	Cas9 protein (1 mg/ml) IDT	NEPA21	4	B6D2F1/Slc (female) x C57BL/6NCrSlc (male) (hybrid)	*Tyr*	4	19	18 (95%)	15 (79%) both alleles
	Tyr-crRNA (30 μM) FASMAC								3 (16%) one allele
	tracrRNA (30 μM) FASMAC								
ssODN knock-in	Cas9 protein (1 mg/ml) IDT	NEPA21	5	C57BL/6NCrl (inbred)	*Cdkn1a*	2	13	5 (38%)	4 (31%)
	crRNA-p21 (30 μM) IDT								
	tracrRNA (30 μM) IDT								
	ssODN-p21 (1 μg/μl) Eurofins Genomics								
ssODN knock-in	Cas9 protein (1 mg/ml) IDT	NEPA21	4	C57BL/6NCrl (inbred)	*Cdkn2a*	2	10	7 (70%)	4 (40%)
	crRNA-p16/p19 (30 μM) IDT								
	tracrRNA (30 μM) IDT								
	ssODN-p16/p19 (1 μg/μl) Eurofins Genomics								
ssODN knock-in	Cas9 protein (1 mg/ml) IDT	CUY21EDIT II	4	BALB/cAJcl (inbred)	*Tyr*	2	6	3 (50%)	1 (17%)
	crRNA-ICR-tyr (30 μM) IDT								
	tracrRNA (30 μM) IDT								
	ssODN-tyr (2 μg/μl) IDT								

### *i*-GONAD using the AsCpf1 nuclease

Recently, Cpf1 derived from *Acidaminococcus sp.* (AsCpf1) has been added as a genome editing tool [22, 23]. We asked whether AsCpf1 protein could be used in *i-*GONAD, which was performed by injecting 6.3 μM of AsCpf1 protein into the oviduct lumen of five pregnant Jcl:MCH(ICR) mice together with 30 μM of crRNA targeting the *Hprt* locus. A total of 40 embryos were isolated at E13.5, and the presence or absence of *indels* was analyzed by PCR and sequencing. DNA from one fetus did not produce a PCR amplicon, probably due to the deletion of primer binding site(s), and DNA from 25 fetuses contained *indel* mutations. The results show that 65% (26/40) of the G0 offspring recovered after *i*-GONAD with AsCpf1 were genome-edited, and about 65% of these samples (17/26) were mosaic (Additional file 1: Table S6).

We also corrected the G308C mutation in the *Tyr* gene using AsCpf1. crRNA was designed for a region spanning the point mutation, and the same ssODN used in the *Tyr* repair experiments described for Cas9-mediated targeting was used (Additional file 1: Figure S7a). *i*-GONAD was performed on four pregnant Jcl:MCH(ICR) females. A total of 19 fetuses were harvested at E13.5. Eleven (58%) of these samples exhibited eye pigmentation indicative of repair (Additional file 1: Figures S7b and S7d). Sequence analysis demonstrated that at least one allele had the corrected sequence (C to G) at the target site in the pigmented fetuses (Additional file 1: Figure S7c), and some fetuses had *indel* mutations. A total of 74% (14/19) of G0 fetuses were genome-edited, when gene correction and *indels* were combined, and the mosaicism frequency was 36% (5/14) (Additional file 1: Table S7). These studies indicate that *i*-GONAD can yield high efficiencies of genome editing when AsCpf1 nuclease substitutes for Cas9 (at least in two loci examined).

### The animals used for *i*-GONAD retain reproductive function

Unlike traditional approaches to genome editing in which female embryo donors are sacrificed to isolate zygotes, the *i*-GONAD method does not require euthanasia of donor females*.* Therefore, we asked whether female mice subjected to *i*-GONAD retain their reproductive function. Three female mice that underwent *i*-GONAD and delivered genome-edited pups (mice-#3, −#5, and -#11 in Additional file 1: Table S2) were mated naturally to fertile male mice. Two of these (67%; mice-#3 and -#11) became pregnant and successfully delivered 9 and 12 pups, respectively (Additional file 1: Figure S8). These data suggest that the oviducts in these mice retained normal function, allowing fertilization and subsequent tubal transport of fertilized eggs to uteri.

## Discussion

### Development of *i-*GONAD

We demonstrated previously a proof-of-principle genome editing method called GONAD [10]. GONAD can be performed on zygotes *in situ* and thus bypasses the steps of isolation of zygotes, their *ex vivo* handling, and their subsequent transfer to recipient females, the steps of animal genome editing that were developed and have been practiced for more than three decades. In this study, we made several improvements to the GONAD method, making it highly suitable for routine creation of genome-edited animal models. First, we assessed the optimal time of pregnancy and showed that day 0.7 is suitable for GONAD, which facilitates introduction of solution into the ampulla. Second, replacing Cas9 mRNA with Cas9 protein and replacing *in vitro* transcribed sgRNA with synthetic components (such as crRNA + tracrRNA) in the approach termed *i*-GONAD enhanced genome editing efficiency up to the levels of microinjection-based approaches. Third, *i*-GONAD can be used for creating large deletion, point mutation, and large cassette knock-in models. Fourth, *i*-GONAD works in many mouse strains. Fifth, the females used for *i*-GONAD retain fully functional reproductive capability. Sixth, the AsCpf1 nuclease can also be used in the *i*-GONAD method. Lastly, we found that *i*-GONAD can be performed using different types of commercially available electroporators. Because electroporators cost ten times less than microinjection setups and do not require specialized personnel, the *i*-GONAD method can be readily adapted by many laboratories that lack one or both.

### Evaluation of timing of the *i-*GONAD procedure

GONAD performed on day 0.7 (corresponding to the late 1-cell stage) was shown to be effective for genome editing. There are several advantages to performing GONAD at this stage. First, the E0.7 zygotes are surrounded by fewer cumulus cells (Fig. 1c) and thus are more easily accessible for delivery of genome editing components (CRISPR-related nucleic acids/protein). We saw that our experiments at day 0.4 did not elicit effective delivery of components via electroporation, probably because zygotes at this stage are surrounded by a cluster of cumulus cells. Second, the oviduct on day 0.7 has a distinctly visible ampulla in many cases, which facilitates micropipette-aided introduction of solutions under a dissecting microscope. Third, in comparison to our previously reported GONAD procedure, which is conducted at day 1.5, the amount of solution introduced at day 0.7 can be reduced to 1.0–1.5 μl per oviduct, which conserves reagents [10, 24]. Fourth, we speculate that because the E0.7 zygotes are in a more compact space within the oviduct ampulla, the genome editing components may reach zygotes more effectively than at day 1.5.

Although we expected less mosaicism with GONAD at day 0.7, mosaicism was still observed, especially when Cas9 mRNA/sgRNA was used (> 82%). The level of mosaicism was considerably lower (~ 36–65%) when ctRNP was used. Very low, or no, mosaicism could potentially be achieved by delivery of CRISPR components at even earlier stages (~ 5 h post-fertilization) [4]. However, performing GONAD at this stage would be quite difficult for two reasons: (1) there are challenges in experimental timing; 5 h post-fertilization is typically very early in the morning for naturally mated mice; and (2) the eggs at this early stage of pregnancy are tightly covered within the cumulus cell complex, which can prevent effective delivery of reagents to zygotes. Nevertheless, mosaicism is not necessarily a major constraint, because most of the genome-edited founders transmit targeted alleles to their offspring.

### Evaluation of different types of CRISPR reagents

Accumulated data from zygote injection and *ex vivo* electroporation-based genome editing show that RNP elicits superior genome editing efficiencies to those achieved using mRNA/sgRNA [7, 13]. In this study, we also found that RNP components directed up to ~ 97% genome editing efficiency, whereas the efficiencies reached using sgRNA/Cas9 mRNA components were only up to 31% (Fig. 2f). Another advantage of the RNP platform is that all components can be commercially made, which will reduce variations resulting from reagents prepared in individual labs. Commercial RNA reagents can be received in lyophilized form for RNA components, and Cas9 protein can be purchased at high concentration. These features allow preparation of electroporation mixes at any desired concentration. For GONAD, the concentration of reagents required in the electroporation mix is typically much higher than the mixes used for direct zygote injection. The fact that 100% of zygotes were genome-edited in some females indicates that the injected electroporation mix successfully surrounded all the zygotes in the oviduct at the time of electroporation.

### *i-*GONAD and *Easi-*CRISPR

The knock-in efficiency of ssODN donors in the *i*-GONAD-treated samples was 49% (Fig. 3e). By using the same combination of locus and genetic modification, we directly compared this efficiency with the microinjection genome editing method, and the efficiency was 52% (Additional file 1: Table S3). The number of animals needed for *i*-GONAD is 2.5 times less than that needed for microinjection-based approaches. One of the limitations of *i*-GONAD is that it requires a higher concentration of reagents than microinjection. Although our data suggest that the efficiency of *i*-GONAD is comparable to that of microinjection, more loci and strains must be tested to assess the comparable efficiencies of the two methods.

As discussed above, *i*-GONAD is a simple and convenient method for production of genome-edited animals. We optimized various parameters of the *i*-GONAD procedure by using the Jcl:MCH(ICR) strain (one of the most fertile mouse strains that produces large litters). We also demonstrated that genome editing by *i*-GONAD works in various mouse strains, although its efficiency is still strain-dependent, and recovery of fetuses/pups in some inbred strains was lower (particularly in C57BL/6), probably because of poor fertility and/or smaller litter sizes in those strains. Thus, further optimization of parameters may be required for some inbred strains. Of note, we recently generated gene-edited rats using *i*-GONAD (Matsuyama et al.: Successful production of genome-edited rats by the rGONAD method, in preparation; Takabayashi et al.: Successful in situ genome editing of rat preimplantation embryos using the improved genome-editing via oviductal nucleic acids delivery (i-GONAD), in preparation), which suggests that the experimental conditions described here can serve as a starting point for applying the method to other mammals.

We also successfully inserted a long ssDNA donor fragment into a target locus using *i*-GONAD. Long ssDNA donors were prepared using the *iv*TRT method as used in our highly efficient knock-in method, “*Easi*-CRISPR” [17–19]. Since a large amount of ssDNA is required for *i*-GONAD, we used spin column-based nucleic acid purification instead of gel purification, where recovery of the sample is poor. Since the microinjection approach does not require high concentrations, gel purification is typically used for zygote-microinjection experiments [17]. The column-purified ssDNA (922–925 bases) exhibited a single band after agarose gel electrophoresis, and it produced knock-in mice when used as the *i*-GONAD donor (Fig. 5). Column-purified long ssDNAs can also be used as donors for creating floxed mice (i.e., mice with a gene locus flanked by *loxP* using *Easi*-CRISPR). Unlike ssODN knock-in, the efficiency of inserting a long donor fragment with the *i*-GONAD method was low (up to 15%) compared with microinjection (25~67%) [18]. However, *i*-GONAD may be superior to microinjection in terms of the number of animals used, because unlike zygote microinjection, maintenance of vasectomized males and production of pseudopregnant females are not required. Thus, *i*-GONAD can be used as an alternative to zygote microinjection for creating knock-in alleles.

### Advantages and applications of *i-*GONAD

Several groups have demonstrated that genome-edited rodents can be produced through *in vitro* electroporation of zygotes [4–9]. The GONAD method goes a step beyond this, given that it directly delivers genome editing nucleic acids and CRISPR components into embryos *in situ*. The GONAD method offers even more advantages over *in vitro* electroporation-based genome editing methods. They are as follows: (1) GONAD does not require *ex vivo* handling of embryos; (2) it does not require *in vitro* cultivation of isolated embryos; (3) it does not require pseudopregnant female mice for implantation of *ex vivo*-treated embryos; (4) it does not require vasectomized males to produce pseudopregnant females (which is particularly advantageous in species where assisted reproductive technologies such as methods of *ex vivo* handling of zygotes and/or methods to prepare surrogate mothers are not well established); and (5) GONAD-treated females need not be sacrificed for zygote isolation. Another very important advantage is that the GONAD-treated females retain reproductive function and can become pregnant again after delivering pups from the GONAD procedure, suggesting that females can be re-used for a second GONAD procedure. This is a very important feature when, e.g., (1) the animals used for GONAD experiments are valuable, and (2) another genetic manipulation can be performed immediately in a newly developed genetically engineered mouse line. This avoids the laborious requirement of expanding the line to produce hundreds of zygotes for performing the second genetic change, as occurs when using microinjection or *ex vivo* electroporation approaches.

We show that *i*-GONAD can be used to rescue pigmentation defects in albino mice (Jcl:MCH(ICR) and BALB/cAJcl strains) and black mice (C57BL/6JJcl strain) by correction of a point mutation in the *Tyr* gene and elimination of a retrotransposon sequence in the *agouti* gene, respectively. Such genetic alterations are quite common in many human genetic diseases [25, 26], and our strategy can be applicable to human germline gene therapy to correct disease-causing mutations. Insertion of long sequences will also be useful in gene therapy strategies based on the addition of a functional gene [27]. Considering that human germline gene therapy will often be coupled with *ex vivo* handling of embryos, including an *in vitro* cell culture step that could cause epigenetic changes to gene expression and affect fetal development [28, 29], *i*-GONAD, which does not require *ex vivo* handling or sacrifice of GONAD-treated females, offers a highly promising approach to human germline gene therapy in the future.

## Conclusions

Animal genome engineering experiments involve three major, but critical, steps: isolation of zygotes from sacrificed females, their micromanipulation *ex vivo*, and then transfer of the treated zygotes into another set of females. These steps have remained largely unchanged for the past four decades. Here we described a new editing method called *i*-GONAD and showed that popular mouse models can be routinely generated without the use of such complex and critical steps. *i*-GONAD offers a number of opportunities that were not possible before. First, *i-*GONAD does not require highly sophisticated equipment or specialized skill sets. This feature is a significant departure from traditional methods, which cannot be performed outside specialized laboratories. Even students or beginner technicians can perform *i*-GONAD. Second, *i-*GONAD uses only 40% or fewer animals than are required by conventional methods. Third, females used in currently used methods will inevitably be euthanized, whereas females used for *i-*GONAD can be recycled; thus, creation of genome-edited animals can occur without loss of the female. These two latter points offer significant benefits from an animal welfare point of view. Fourth, the *i-*GONAD method established in this study can be readily adapted for genome editing in other mammals such as the rat, other rodents, primates, and large animals. The method is particularly powerful for rare and valuable animals which cannot be sacrificed for zygote collection and/or for animals in which *ex vivo* handling of zygotes has not been established. Lastly, *i*-GONAD-treated females fully retain reproductive function; thus, the approach holds high promise as an *in vivo* gene therapy tool for germline gene correction.

## Methods

### CRISPR reagents

CRISPR guide RNAs were designed using CRISPR.mit.edu or CHOPCHOP (Additional file 1: Table S8). The sgRNA for *Foxe3* was synthesized as described previously [10] using the primer sets (M1055/M939) and the pUC57-sgRNA vector as a template (Addgene plasmid number: #51132). The mRNAs for eGFP and Cas9 were *in vitro* transcribed as previously described [10, 24]. The synthetic crRNA and tracrRNA were commercially obtained as Alt-R™ CRISPR guide RNAs from Integrated DNA Technologies (IDT), Skokie, IL, USA or purchased from FASMAC, Kanagawa, Japan together with Cas9 protein (Alt-R™ S.p. CAS9 Nuclease 3NLS). The ssODN donors were custom synthesized from IDT (Ultramer: for *Tyr* rescue experiment [*Tyr*-rescue] and *agouti* rescue experiment [*agouti*-rescue]) or synthesized from Eurofins Genomics, Louisville, KY, USA (for ssODN knock-in into the *Cdkn1a* and *Cdkn2a* genes). Long ssDNA donors (for *Pitx3* and *Tis21* reporters) were prepared from the double-stranded DNA (dsDNA) templates using the *iv*TRT method described previously [17] with slight modifications. The T2A-mCitrine cassette was amplified from the original vector (pP200) with primer sets (M1051/M1052 for *Pitx3* and M1053/M1054 for *Tis21*) and inserted into the *Sma*I site of pUC119, resulting in pP206 (for *Pitx3*) and pP209 (for *Tis21*). The templates for RNA synthesis were amplified from these vectors with primer sets (PP226/M272 for *Pitx3* and PP227/M272 for *Tis21*), and RNAs were synthesized using the T7 RiboMax Express Large Scale RNA Production System (Promega, Madison, WI, USA). The RNAs were purified using a MEGAclear Kit (Ambion), and the cDNAs were generated using SuperScript III Reverse Transcriptase (for *Pitx3*) or SuperScript IV Reverse Transcriptase (for *Tis21*; Life Technologies) with the primers PP226 for *Pitx3* and PP227 for *Tis21*. The final step of gel extraction, as done for purifying cDNA for microinjection, was excluded in order to obtain a sufficiently higher concentration of the final ssDNA. Instead, spin column-based nucleic acid purification using NucleoSpin Gel and PCR Clean-up (Macherey-Nagel, Düren, Germany) was performed. After ethanol precipitation, the DNA pellet was dissolved in EmbryoMax Injection Buffer (Millipore). The sequences for primers and ssODNs are shown in Additional file 1: Table S9.

### Animals

Mice were maintained at the animal facility in Tokai University School of Medicine, Hamamatsu University School of Medicine, or Shigei Medical Research Institute. Adult Jcl:MCH(ICR) (hybrid strain originally derived from Jcl:ICR strain: http://www.clea-japan.com/en/animals/animal_g/g_01.html), C57BL/6JJcl (inbred strain), and BALB/cAJcl (inbred strain) mice were obtained from CLEA Japan, Inc. (Tokyo, Japan); C3H*/*HeSlc (inbred strain), C57BL/6NCrSlc (inbred strain), DBA/2CrSlc (inbred strain), and B6D2F1/Slc (hybrid strain) mice were obtained from Japan SLC, Inc. (Shizuoka, Japan); and C57BL/6NCrl (inbred strain) mice were obtained from Charles River Laboratories Japan, Inc. (Yokohama, Japan). All the animal experiments were performed in accordance with institutional guidelines and were approved by the Institutional Animal Care and Use Committee (Permit Numbers #154014, #165009, #171003 at Tokai University, #2017062 at Hamamatsu University, and #17008 at Shigei Medical Research Institute).

### Preparation of CRISPR electroporation solutions

The solution contained *in vitro* synthesized sgRNAs (or commericailly procured crRNA/tracrRNA mixes) and the commercially procured Cas9 protein. When the donor DNAs were included in the electropoartion solutions, they were either commercially synthesized ssODNs or *iv*TRT synthesized long ssDNAs. The Cas9 mRNA/sgRNA mixture was prepared as we previously described [10]. We used 0.05% of trypan blue (Nacalai Tesque Inc., Kyoto, Japan) as a marker for successful injection, only when eGFP mRNA was used or Cas9 was supplied as mRNA. Lyophilized ssODNs were re-suspended in nuclease-free water to a concentration of 10 μg/μl. Lyophilized crRNA and tracrRNA were first re-suspended in RNase-free Duplex Buffer to a concentration of 200 μM. Equal volumes of crRNA and tracrRNA were combined in a 1.5-ml tube, heated in a thermocycler to 94 °C for 2 min, and then placed at room temperature for about 10 min. The annealed crRNA and tracrRNA were mixed with Cas9 protein and/or ssODN/ssDNA so that the final concentrations of components were 30 μM (for crRNA/tracrRNA), 1 mg/ml (for Cas9 protein), 1 or 2 μg/μl (for ssODN), and 0.85~1.4 μg/μl (for ssDNA). AsCpf1 crRNA (MmHPRT-273-S: 5’-GTGCCCTCTTCTGGCCTGCCA-3′) was a kind gift from IDT. Lyophilized crRNAs were first re-suspended in RNase-free water to a concentration of 100 μM and then heated in a thermocycler to 95 °C for 5 min and placed at room temperature for about 10 min. AsCpf1 protein (IDT) was mixed with crRNA so that the final concentrations of components were 30 μM (for crRNA) and 6.3 μM (for AsCpf1 protein). The electroporation solution was occasionally diluted using Opti-MEM (Thermo Fisher Scientific) to adjust the volume to 1.5 μl/oviduct.

### GONAD procedure

The females used for the procedure were not super-ovulated except for the C57BL/6JJcl strain. For all of the strains except C57BL/6JJcl, females in estrus were mated with stud males. Matings were set up at 16:00–17:00, and copulation plugs were confirmed by visual inspection the next morning (9:00–10:00). We designated day 0 of gestation at 0:00 (midnight) according to *Manipulating the Mouse Embryo: A Laboratory Manual* [30], and the females with plugs were designated as day 0.4 of gestation at 10:00 and day 0.7 of gestation at 16:00, at which time they were used for the electroporation experiments.

Surgical procedures were performed on anesthetized females at day 0.7 of pregnancy (corresponding to late 1-cell stage zygotes, at 16:00 of the same day when the plugs were confirmed) under observation using a dissecting microscope (SZ11; Olympus, Tokyo, Japan), as described previously [10, 24] with slight modifications. The ovary/oviduct/uterus was exposed after making an incision at the dorsal skin. Approximately 1.0–1.5 μl of electroporation solution (pre-warmed at 37°C for 10 min) was injected into the oviduct lumen from upstream of the ampulla using a micropipette. The micropipette apparatus consisted of a glass capillary needle (pulled using a P-97/IVF electric puller; Sutter Instrument Co., Novato, CA, USA) and a mouthpiece attached to the needle. Immediately after the injection of solution, the oviduct regions were covered with a piece of wet paper (Kimwipe; Jujo-Kimberly Co. Ltd., Tokyo, Japan) soaked in phosphate-buffered saline (PBS) and then grasped in tweezer-type electrodes (CUY652–3 [NEPA GENE Co. Ltd., Ichikawa, Chiba, Japan] for T820 and NEPA21, and LF650P3 [BEX Co. Ltd., Tokyo, Japan] for CUY21EDIT II). The electroporation was performed using a square-wave pulse generator T820 (BTX Genetronics Inc.), or NEPA21 (NEPA GENE), or CUY21EDIT II (BEX). The electroporation parameters were as follows: eight square-wave pulses with a pulse duration of 5 ms, a pulse interval of 1 s, and an electric field intensity of 50 V for T820; poring pulse: 50 V, 5-ms pulse, 50-ms pulse interval, 3 pulse, 10% decay (± pulse orientation) and transfer pulse: 10 V, 50-ms pulse, 50-ms pulse interval, 3 pulse, 40% decay (± pulse orientation) for NEPA21; and square (mA), (+/−), Pd V: 60 V or 80 V, Pd A: 200 mA, Pd on: 5.00 ms, Pd off: 50 ms, Pd N: 3, Decay: 10%, DecayType: Log for CUY21EDIT II. After the electroporation, the oviducts were returned to their original position, and the incisions were sutured. The animals were monitored for anesthesia recovery and were housed for further analysis.

### Microinjection

CRISPR components were mixed in EmbryoMax Injection Buffer. The final concentrations of Cas9 protein, crRNA/tracrRNA, and ssODN (for *Tyr* rescue) were 50 ng/μl, 0.61 μM, and 10 ng/μl, respectively. Unfertilized oocytes isolated from super-ovulated female mice (Jcl:MCH(ICR)) were subjected to *in vitro* fertilization (IVF) with spermatozoa freshly isolated from a Jcl:MCH(ICR) male mouse. Microinjection of the mixture was performed into pronuclei of *in vitro* fertilized eggs. The injected embryos were transferred into the oviduct of pseudopregnant Jcl:MCH(ICR) females to allow further development. The resulting fetuses (day 13.5 or 15.5) were recovered and subjected to genotyping analysis.

### Observation of mCitrine fluorescence

The fetuses recovered were observed using a fluorescence stereomicroscope with filter for GFP (Olympus SZX7 with SZX-MGFPA) for detecting the mCitrine fluorescence.

### Analysis of CRISPR/Cas9-induced mutations and insertions

Genomic DNAs were isolated from the limb of mid-gestational fetuses or the ear-piece of live mice using All-In-One Mouse Tail Lysis Buffer (ABP-PP-MT01500; Kurabo, Osaka, Japan) through incubation at 55 °C for 3 h or overnight and subsequent inactivation at 85 °C for 45 min. The PCR for amplification of target loci *Foxe3*, *Tyr*, *agouti*, and *Hprt* was performed in a total of 10 μl solution containing 5 μl of 2 × GC buffer I, 0.2 mM deoxynucleotide (dNTP), 1 μl of the crude lysate, the primer pairs (Additional file 1: Table S9), and 0.125 U of TaKaRa r-Taq (TaKaRa) using denaturation (95 °C for 5 min), 35 cycles of 95 °C for 45 s, 58 °C for 30 s, and 72 °C for 1 min, and extension (72 °C for 5 min). For amplification of target loci *Pitx3* and *Tis21*, PCR amplifications were performed using PrimeSTAR HS DNA Polymerase (TaKaRa) in a total of 10 μl solution containing 2 μl of 5 × PrimeSTAR buffer I, 0.2 mM dNTP, 1 μl of the crude lysate, the primer pairs (Additional file 1: Table S9), and 0.25 U of PrimeSTAR HS DNA Polymerase using denaturation (94 °C for 3 min), 35 cycles of 98 °C for 10 s, 62 °C or 64 °C for 5 s, and 72 °C for 2 min, and extension (72 °C for 10 min). Direct sequencing was performed using the PCR products and the primers listed in Additional file 1: Table S9.

Mosaicism of alleles was assessed by observing an electropherogram of Sanger sequence results. Mosaicism was assessed based on the following criteria: (1) the presence of multiple peaks consisting of more than three peaks (or two peaks in the *Hprt* gene of male mice) and (2) one of the two overlapping peaks apparently lower or higher than the other. Determination of gender was performed with PCR using primer set Sry-F2 and Sry-R2 (Additional file 1: Table S9) [31].

## Additional file

Additional file 1: **Figure S1.** Fetuses recovered from the *i*-GONAD procedure to edit the *Tyr* gene. **Figure S2.** Germline transmission of *Tyr*-gene-corrected allele. **Figure S3.** Generation of reporter knock-in mice at the *Tis21* locus using the GONAD method. **Figure S4.** Generation of *indel* mutation in the *Tyr* locus of various mouse strains using *i*-GONAD. **Figure S5.** Generation of *indel* mutation in the *Kit* locus of C3H/HeSlc and C57BL/6NCrSlc mouse strains using *i*-GONAD. **Figure S6.** Knock-in of ssODN into *Cdkn1a* and *Cdkn2a* loci in the C57BL/6NCrl mouse strain using *i*-GONAD. **Figure S7.** Restoration of *Tyr* mutation of albino Jcl:MCH(ICR) mice by ssODN-based knock-in using *i*-GONAD with AsCpf1. **Figure S8.** *i*-GONAD-used females retain reproductive capability. **Table S1.** Generation of *Foxe3* knock-out mice using conventional GONAD and *i*-GONAD approaches. **Table S2.** Correction of *Tyr* mutation by ssODN knock-in using the *i*-GONAD method. **Table S3.** Correction of *Tyr* mutation by zygote microinjection of CRISPR/Cas9 components. **Table S4.** Restoration of *agouti* gene expression by elimination of retrotransposon sequence using the *i-*GONAD method. **Table S5.** Generation of reporter gene knock-in mice using *i*-GONAD with ssDNA as donors. **Table S6.** Editing of the *Hprt* locus using *i*-GONAD with AsCpf1. **Table S7.** Correction of *Tyr* mutation using the *i*-GONAD with AsCpf1. **Table S8.** CRISPR target sequences and the types of gRNA used. **Table S9.** Sequences of the oligonucleotides used in this study. (PDF 7440 kb)
