# Supplementary material for: i-GONAD: a robust method for in situ germline genome engineering using CRISPR nucleases
Source: Genome Biol. 2018 Feb 26;19:25. doi: 10.1186/s13059-018-1400-x (PMC5828090; doi:10.1186/s13059-018-1400-x)
Supplement: Supplementary file 1 — Figure S1. Fetuses recovered from the i-GONAD procedure to edit the Tyr gene. Figure S2. Germline transmission of Tyr-gene-corrected allele. Figure S3. Generation of reporter knock-in mice at the Tis21 locus using the GONAD method. Figure S4. Generation of indel mutation in the Tyr locus of various mouse strains using i-GONAD. Figure S5. Generation of indel mutation in the Kit locus of C3H/HeSlc and C57BL/6NCrSlc mouse strains using i-GONAD. Figure S6. Knock-in of ssODN into Cdkn1a and Cdkn2a loci in the C57BL/6NCrl mouse strain using i-GONAD. Figure S7. Restoration of Tyr mutation of albino Jcl:MCH(ICR) mice by ssODN-based knock-in using i-GONAD with AsCpf1. Figure S8. i-GONAD-used females retain reproductive capability. Table S1. Generation of Foxe3 knock-out mice using conventional GONAD and i-GONAD approaches. Table S2. Correction of Tyr mutation by ssODN knock-in using the i-GONAD method. Table S3. Correction of Tyr mutation by zygote microinjection of CRISPR/Cas9 components. Table S4. Restoration of agouti gene expression by elimination of retrotransposon sequence using the i-GONAD method. Table S5. Generation of reporter gene knock-in mice using i-GONAD with ssDNA as donors. Table S6. Editing of the Hprt locus using i-GONAD with AsCpf1. Table S7. Correction of Tyr mutation using the i-GONAD with AsCpf1. Table S8. CRISPR target sequences and the types of gRNA used. Table S9. Sequences of the oligonucleotides used in this study. (PDF 7440 kb) [file 13059_2018_1400_MOESM1_ESM.pdf]

Additional file 1: Supplementary Figures and Tables

Supplementary Figures:
Figure S1; Fetuses recovered from the *i*-GONAD procedure to edit the *Tyr*-gene.
Figure S2; Germline transmission of *Tyr*-gene-corrected allele.
Figure S3; Generation of reporter knock-in mice at the *Tis21* locus using the GONAD method.
Figure S4; Generation of *indel* mutation in the *Tyr* locus of various mouse strains using *i*-GONAD.
Figure S5; Generation of *indel* mutation in the *Kit* locus of C3H/HeSlc and C57BL/6NCrSlc mouse strains using *i*-GONAD.
Figure S6; Knock-in of ssODN into *Cdkn1a* and *Cdkn2a* loci in the C57BL/6NCrl mouse strain using *i*-GONAD.
Figure S7; Restoration of *Tyr* mutation of albino Jcl:MCH(ICR) mice by ssODN-based knock-in using *i*-GONAD with AsCpf1.
Figure S8; *i*-GONAD-used females retain reproductive capability.

Supplementary Tables:
Table S1; Generation of *Foxe3* knock-out mice using conventional GONAD and *i*-GONAD approaches.
Table S2; Correction of *Tyr* mutation by ssODN knock-in using the *i*-GONAD method.
Table S3; Correction of *Tyr* mutation by zygote microinjection of CRISPR/Cas9 components.
Table S4; Restoration of *agouti* gene expression by elimination of retrotransposon sequence using the *i*-GONAD method.
Table S5; Generation of reporter gene knock-in mice using *i*-GONAD with ssDNA as donors.
Table S6; Editing of the *Hprt* locus using *i*-GONAD with AsCpf1.
Table S7; Correction of *Tyr* mutation using the *i*-GONAD with AsCpf1.
Table S8; CRSIPR target sequences and the types of gRNA used.
Table S9; Sequences of the oligonucleotides used in this study.

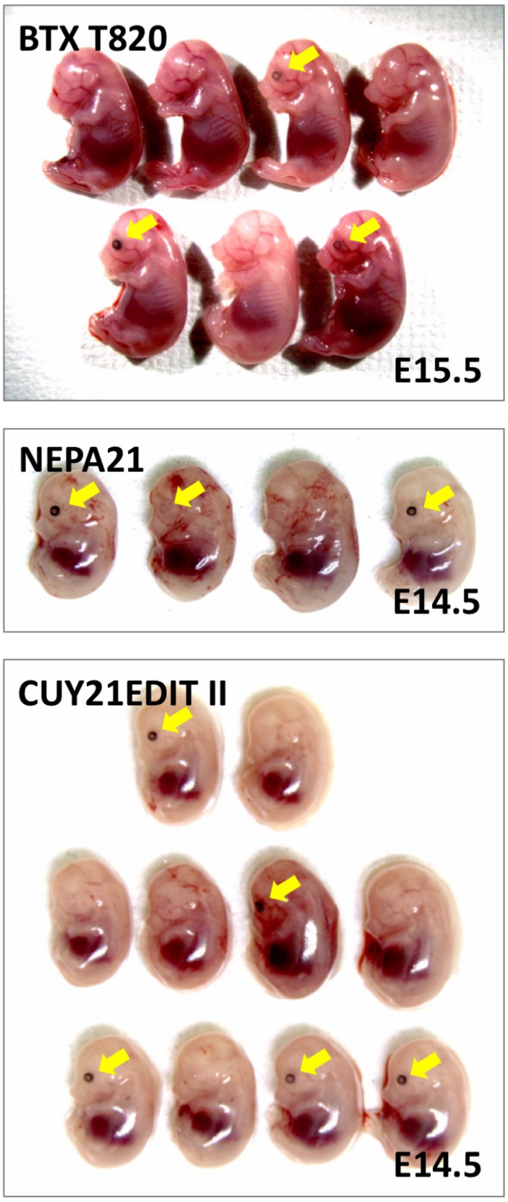

Figure S1. Fetuses recovered from the *i*-GONAD procedure to edit the *Tyr*-gene. Data shows that *i*-GONAD can produce comparable levels of genome editing using electroporators from three different manufacturers. The pigmented eyes of the fetuses are indicated by yellow arrows.

a

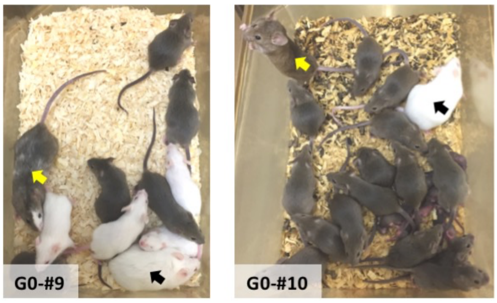

b

G0 mice	No. G1 newborns obtained	No. G1 newborns with agouti coat color
#8	43	0
#9	18	8
#10	30	30

c

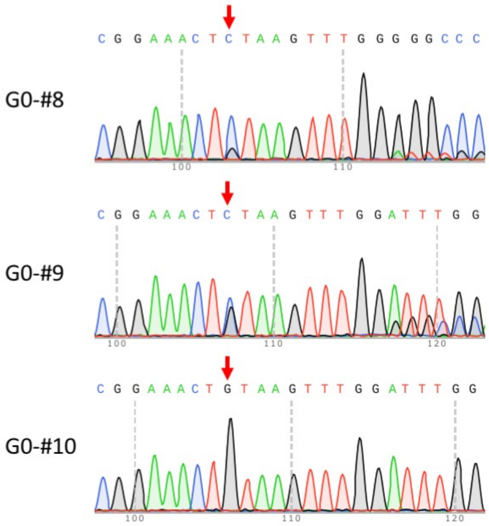

Figure S2 Germline transmission of *Tyr*-gene-corrected allele. The G0 mice (G0-#8, -#9, and -#10 obtained from female #5 mouse in **Table S2**; see also **Fig. 3c**) were mated with Jcl:MCH(ICR) mice and germline transmission of repaired allele was checked by coat color observation. **a** The G0-#9 and -#10 mice (shown in yellow arrows) were mated with Jcl:MCH(ICR) mice (shown in black arrows) to obtain G1 offspring. **b** Germline transmission rate. **c** Direct sequencing results of PCR products amplified from each of the G0 mouse. Red arrows indicate the position of the mutated/corrected nucleotides.

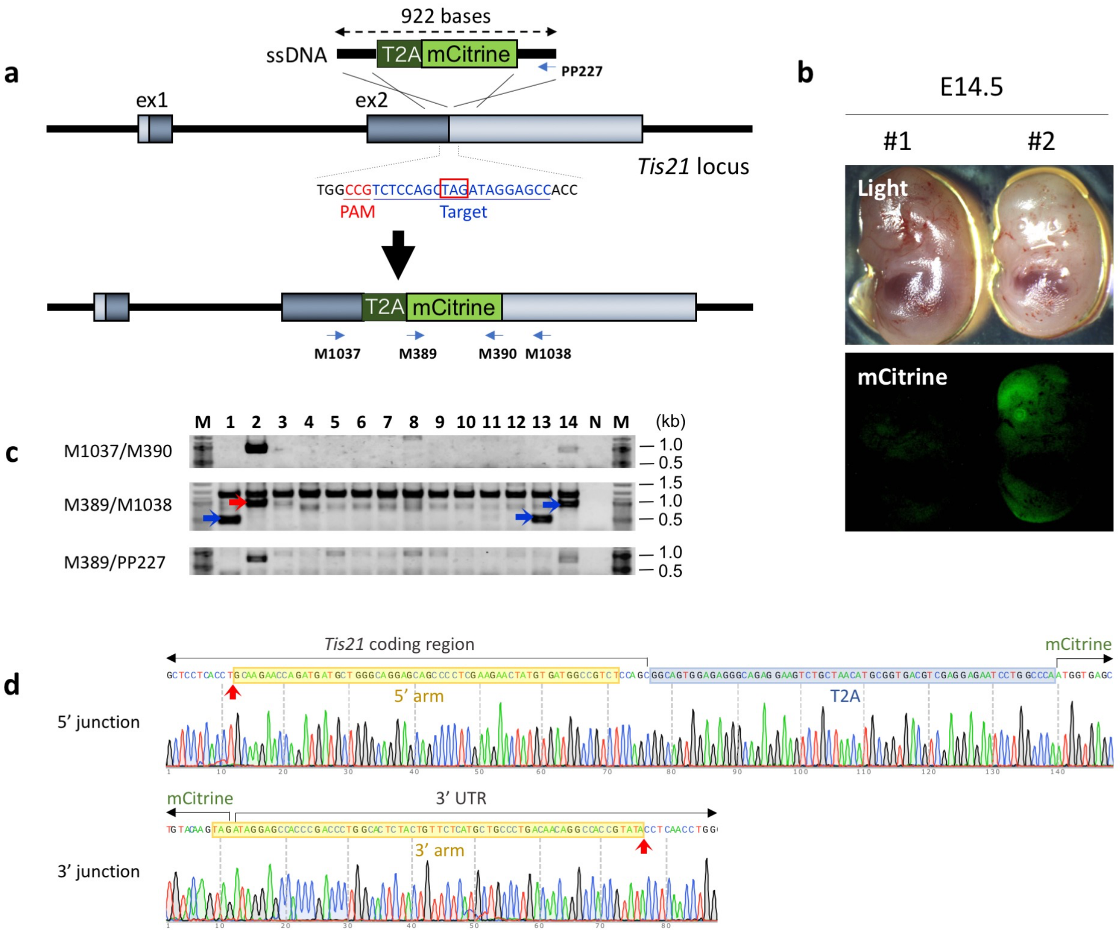

e

No. mice treated	Concentration of ssDNA	No. G0 fetuses/newborns obtained	No. G0 mice with modified allele	No. G0 mice with knock-in allele
2	0.85 µg/µl	14	5 (36%)	1 (7%)

Figure S3 Generation of reporter knock-in mice at the *Tis21* locus using the *i*-GONAD method. **a** Schematic diagram illustrating the insertion of "T2A-mCitrine" cassette into the *Tis21* locus. The target sequence and the primer sets used for genotyping are shown. A 922 bases-long ssDNA synthesized by *iv*TRT method was used as the donor DNA. **b** mCitrine fluorescence in the G0-#2 fetus collected at Day 14.5 (right) whereas no fluorescence was detected in the fetus G0-#1 (left). **c** Genotyping analysis for the knock-in allele in G0 fetuses. Expected fragment sizes: M1037/M390 = 964-bp, M389/M1038 = 1005-bp, M389/PP227 = 802-bp. N: negative control, M: size marker. The PCR band shown with a red arrow indicates the correct insertion of the cassette and the bands shown in blue arrows indicate partial insertion. **d** sequencing chromatogram showing 5' and 3' junctional regions of the inserted cassette. The junctional sequences showing insertion in the G0-#2 fetus in **b** is shown. Red arrows indicate junctions between the arms and the genomic sequences. **e** Genome editing efficiency of the *Tis21* locus using the *i*-GONAD method.

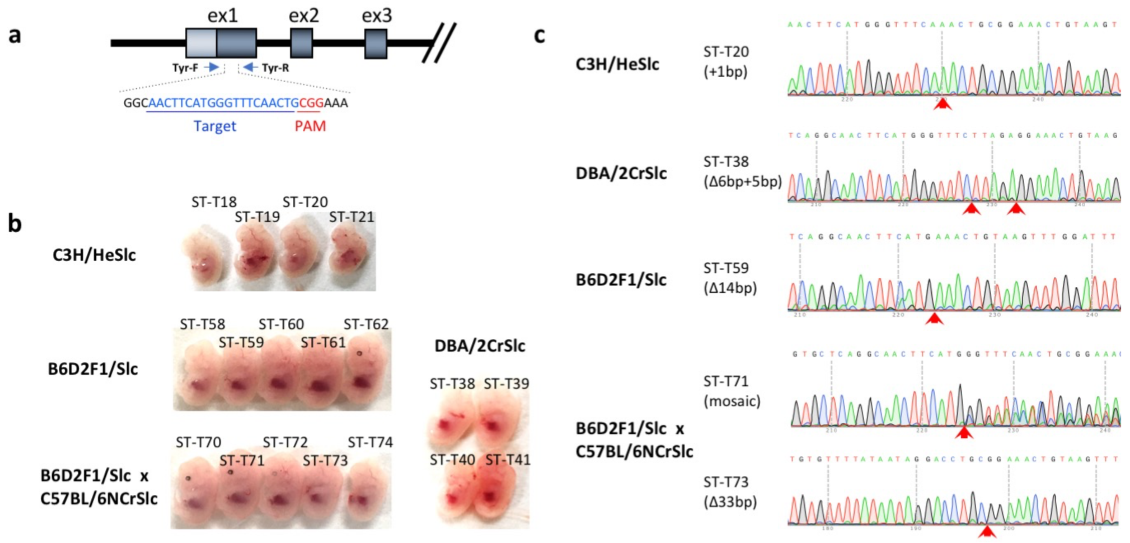

Figure S4 Generation of *indel* mutation in the *Tyr* locus of various mouse strains using *i*-GONAD. **a** Schematic of the *Tyr* gene targeting strategy and the primer set used for genotyping. **b** Representative fetuses showing *Tyr* knockout phenotype (loss of eye pigmentation). **c** Direct sequencing results of PCR products amplified from each of the G0 fetuses. Red arrows indicate the position of the mutated nucleotides.

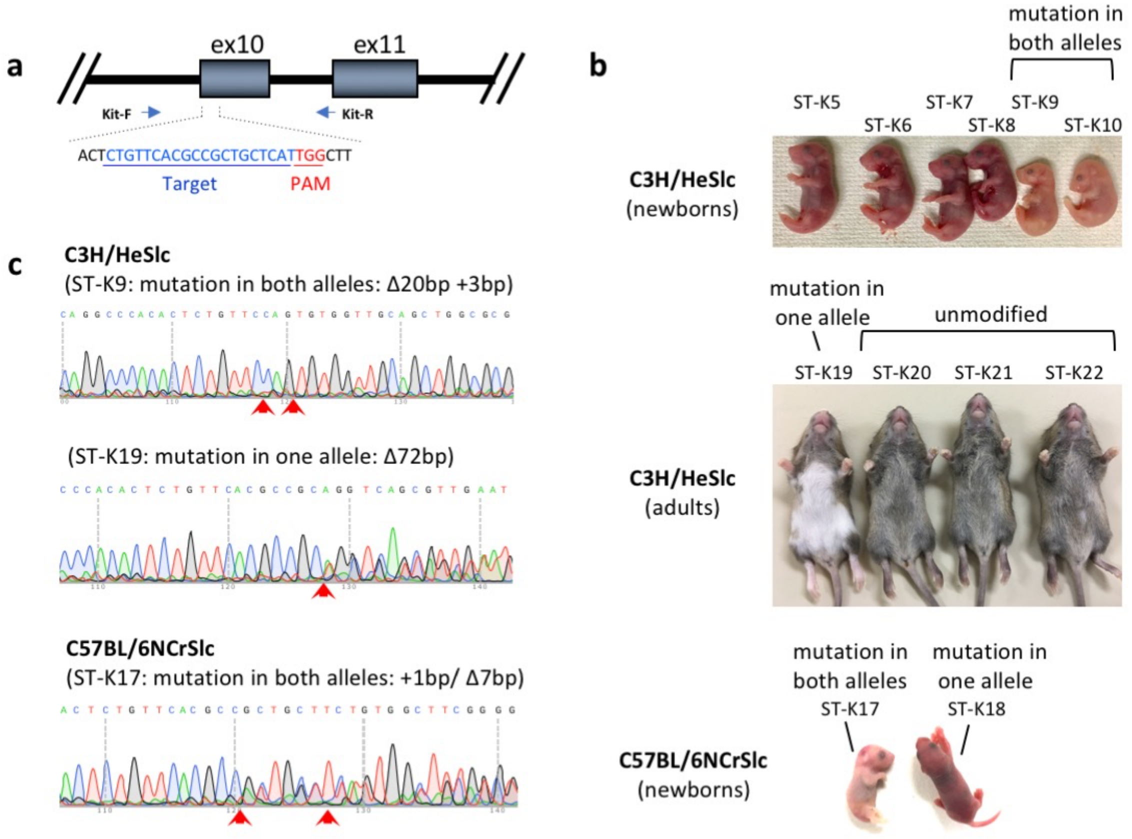

Figure S5 Generation of *indel* mutation in the *Kit* locus of C3H/HeSlc and C57BL/6NCrSlc mouse strains using *i*-GONAD. **a** Schematic of the *Kit* gene targeting strategy and the primer set used for genotyping. **b** Representative fetuses showing *Kit* knockout phenotype (body color phenotype). **c** Direct sequencing results of PCR products amplified from each of the G0 fetuses. Red arrows indicate the position of the mutated nucleotides.

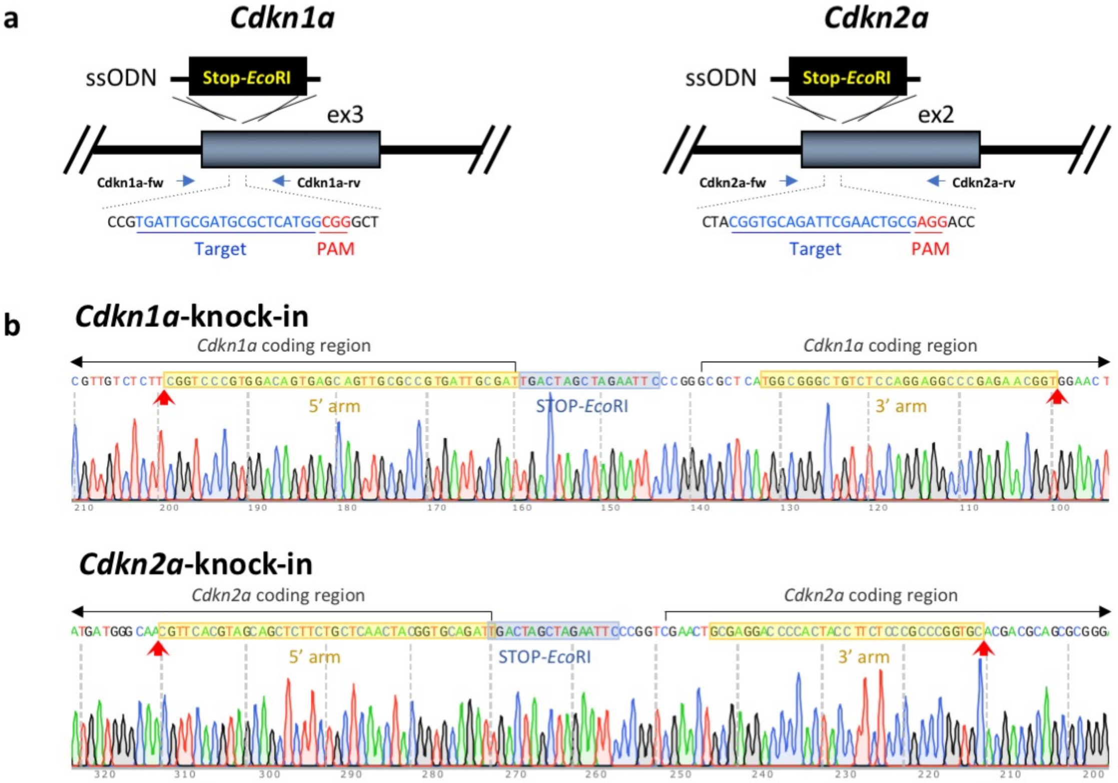

Figure S6 Knock-in of ssODN into *Cdkn1a* and *Cdkn2a* loci in the C57BL/6NCrl mouse strain using *i*-GONAD. **a** Schematic of the targeting strategy to inactivate *Cdkn1a* or *Cdkn2a* gene and the primer sets used for genotyping. **b** Sequencing results of cloned PCR products amplified from each of the G0 fetuses. Red arrows indicate junctions between the arms and the genomic sequences.

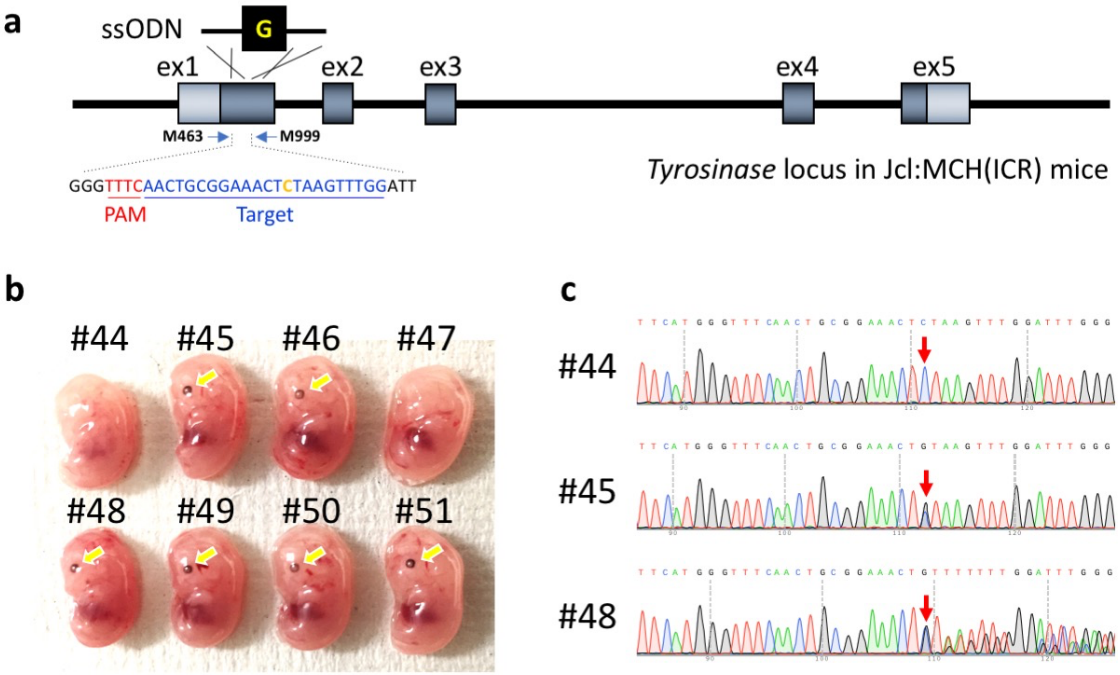

d

No. mice treated	Electroporator used	No. G0 fetuses obtained	No. G0 mice with modified allele	No. G0 mice with repaired allele
4	CUY21EDIT II	19	14 (74%)	11 (58%)

Figure S7 Restoration of *Tyr* mutation of albino Jcl:MCH(ICR) mice by ssODN-based knock-in using *i*-GONAD with AsCpf1. **a** Schematic to show rescue of *Tyr* gene mutation. The target region containing the guide sequence for AsCpf1 and the genotyping primer binding sites are shown. **b** Representative E14.5 litter showing *Tyr* rescued G0 fetuses. The pigmented eyes of the fetuses are indicated by yellow arrows. **c** Direct sequencing results of PCR products amplified from the G0 fetuses (in **b**). The position for mutated/corrected nucleotides are indicated by red arrows. **d** Efficiencies of *Tyr* gene editing.

a

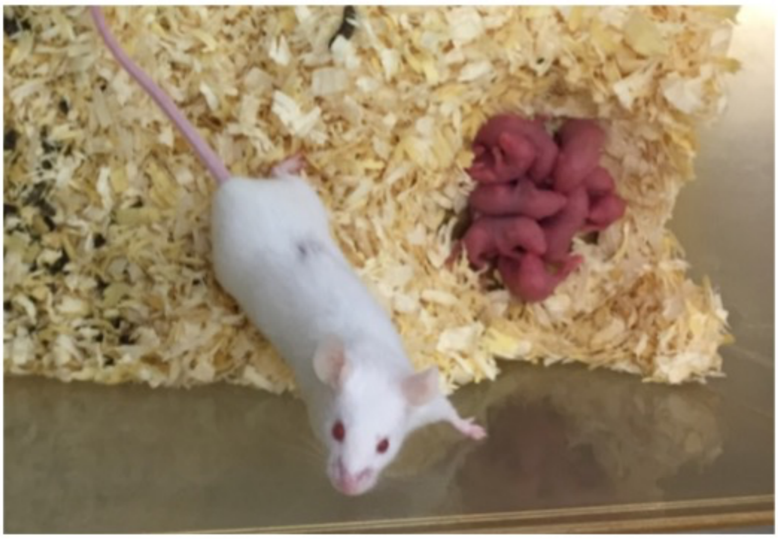

b

Female mice used for *i*-GONAD	No. pups delivered
#3	9
#5	0
#11	12

Figure S8 *i*-GONAD-used females retain reproductive capability. *i*-GONAD-used female mice (#3, #5, and #11 in **Table S2**) were mated with Jcl:MCH(ICR) males in their next cycle. **a** Representative *i*-GONAD-used female mouse #3 with her litter of 9 pups. **b** Litter size of all three *i*-GONAD-used females.

Table S1. Generation of *Foxe3* knock-out mice using conventional GONAD and *i*-GONAD approaches.

Mice	Concentration of CRISPR/Cas9 components	Electroporator used	No. of G0 fetuses/pups obtained	No. of G0 fetuses/pups with mutation	No. of mosaic fetuses/pups
#1	**conventional GONAD**<br>Cas9 mRNA (1.5µg/µl)<br>Foxe3_3_Cr_sgRNA (0.8µg/µl)	BTX T820	7	4	4
#2					
#3			13	3	3
#4					
#5			11	3	2
#6					
#7			5	1	0
#8					
total			36	11 (31%)	9 (82%)
#9	***i*-GONAD**<br>Cas9 protein (1mg/ml)<br>crRNA-Foxe3-3-Cr (30µM)<br>tracrRNA (30µM)	BTX T820	2	2	1
#10			2	2	1
#11			9	9	7
#12		CUY21EDIT II	6	5	2
#13			5	5	4
#14			3	3	0
#15			9	9	5
total			36	35 (97%)	20 (57%)

Table S2. Correction of *Tyr* mutation by ssODN knock-in using the *i*-GONAD method.

**Female mice**	**Concentration of CRISPR/Cas9 components**	**Electroporator used**	**No. of G0 fetuses/ pups obtained**	**No. of G0 fetuses/pups with modified allele**	**No. of G0 fetuses/pups with repaired allele**	**No. of G0 fetuses/pups with *indel* mutation**	**No. of mosaic fetuses/pups**
#1	Cas9 protein (1mg/ml)<br>crRNA-ICR-tyr (30µM)<br>tracrRNA (30µM)<br>ssODN-tyr (2µg/µl)	BTX T820	4	4	1	4	0
#2			7	7	3	7	5
#3			4	4	1	4	2
#4			4	3	3	3	2
#5			13	11	7	10	9
total			32	29 (91%)	15 (47%)	28 (88%)	18 (62%)
#6		NEPA21	7	7	4	6	3
#7			10	7	3	7	5
#8			4	4	4	2	2
#9			4	4	2	4	2
#10			0	0	0	0	0
#11			7	5	3	5	3
total			32	27 (84%)	16 (50%)	24 (75%)	15 (56%)
#12		CUY21EDIT II	6	6	2	6	5
#13			4	4	3	2	2
total			10	10 (100%)	5 (50%)	8 (80%)	7 (70%)
total of all the experiments			74	66 (89%)	36 (49%)	60 (81%)	40 (61%)

Table S3. Correction of *Tyr* mutation by zygote microinjection of CRISPR/Cas9 components.

**Exp.**	**Eggs injected**	**Embryos transferred**	**No. of G0 fetuses obtained**	**No. of G0 fetuses with modified allele**	**No. of G0 fetuses with repaired allele**	**No. of G0 fetuses with *indel* mutation**	**No. of mosaic fetuses**
1	64	50	3	1	1	0	0
2	116	93	32	24	17	16	8
3	159	99	27	24	14	19	13
total	339	242	62	49 (79%)	32 (52%)	35 (56%)	21 (43%)

Table S4. Restoration of *agouti* gene expression by elimination of retrotransposon sequence using the *i*-GONAD method.

Female mice	Concentration of CRISPR/Cas9 components	Electroporator used	No. of G0 mice obtained	No. of G0 mice with modified allele	No. of G0 mice with repaired allele	Knock-in of ssODN
#3	Cas9 protein (1mg/ml)<br>Agouti-crRNA-1 (15µM)<br>Agouti-crRNA-2 (15µM)<br>tracrRNA (30µM)<br>ssODN (2µg/µl)	BTX T820	2	0	0	0
#4			0	0	0	0
#7			0	0	0	0
#8			0	0	0	0
#1		NEPA21	1	1	1	1
#2			0	0	0	0
#5			1	1	1	0
#6			2	2	1	0
total			6	4 (67%)	3 (50%)	1 (17%)

Table S5. Generation of reporter gene knock-in mice using *i*-GONAD with ssDNA as donors.

Female mice	Locus	Concentration of ssDNA	No. of G0 fetuses obtained	No. of G0 fetuses with modified allele*	No. of G0 knock-in fetuses
#1	*Pitx3*	1.3μg/μl	8	5	1
#2			13	6	1
#3		1.4μg/μl	13	10	3
total			34	21 (62%)	5 (15%)
#4	*Tis21*	0.85μg/μl	11	2	1
#5			3	3	0
total			14	5 (36%)	1 (7%)

* including those containing *indel* mutation, complete knock-in cassette, and partial knock-in.

Table S6. Editing of the *Hprt* locus using *i*-GONAD with AsCpf1.

Female mice	Concentration of Cpf1/crRNA components [Electroporator used]	No. of G0 fetuses obtained	No. of G0 fetuses with mutation	No. of mosaic fetuses
#1	Cpf1 protein (6.3μM)<br>Cpf1 crRNA (30μM)<br><br>[NEPA21]	2	2	2
#2		9	9	6
#3		6	4	1
#4		16	7	5
#5		7	4	3
total		40	26 (65%)	17 (65%)

Table S7. Correction of *Tyr* mutation using the *i*-GONAD with AsCpf1.

**Female mice**	**Concentration of AsCpf1/crRNA components [Electroporator used]**	**No. of G0 fetuses obtained**	**No. of fetuses with modified allele**	**No. of fetuses with repaired allele**	**No. of fetuses with *indel* mutation**	**No. of mosaic fetuses**
#1	AsCpf1 protein (6.3 µM)	6	6	3	4	3
#2	AsCpf1 crRNA-ICR-tyr (30 µM)	5	2	2	0	0
#3	ssODN-tyr (2µg/µl)	8	6	6	3	2
#4	[CUY21EDIT II]	0	0	0	0	0
total		19	14 (74%)	11 (58%)	7 (37%)	5 (36%)

Table S8. CRSIPR target sequences and the types of gRNA used.

**Target loci**	**Target seqs (5'-3')**	**PAM**	**Types of gRNA used**
Foxe3	GAGACAGCCGGGCTTCGCGC	CGG	crRNA/tracrRNA or sgRNA
Tyr (ICR)	GGAAACTCTAAGTTTGGATT	TGG	crRNA/tracrRNA
Agouti (1)	AATGGACATTTAGTCGAACT	GGG	crRNA/tracrRNA
Agouti (2)	AGGGTTTAACCACCTATCGA	AGG	crRNA/tracrRNA
Pitx3	CGGTGTGAGCCGCAGGTCTG	TGG	crRNA/tracrRNA
Tis21	GGCTCCTATCTAGCTGGAGA	CGG	crRNA/tracrRNA
Tyr (wild)	AACTTCATGGGTTTCAACTG	CGG	crRNA/tracrRNA
Kit	CTGTTCACGCCGCTGCTCAT	TGG	crRNA/tracrRNA
p21 (Cdkn1a)	TGATTGCGATGCGCTCATGG	CGG	crRNA/tracrRNA
p16/p19 (Cdkn2a)	CGGTGCAGATTCGAACTGCG	AGG	crRNA/tracrRNA

Table S9. Sequences of the oligonucleotides used in this study.

Name of oligos	Sequences (5'-3')
Tyr-rescue*	TGTTTTATAATAGGACCTGCCAGTGCTCAGGCAACTTCATGGGTTTCAACTGCGGAAACTGTAAGTTTGGATTTGGGGGCCCAAATTGTACAGAGAAGCGAGTCTTGATTAGAAGAAACATTTTTGATTTG
agouti-rescue*	TTTATTGCAACCTGCCTTTGCCTTTATATGTGTTGAATATTTTTAGACTTGATACCCAGTGAATTCGAAGGGTTTTCCCAAACCCCTCCTCAGAACTCAGGAGTATCATTAAGGTACTGCGGTTT
M1035	TCCTCCCCCTATGTATACCG
M1036	TCCCTGTTCCTGGCCTTAG
M1037	CCTGTGGGTTGATCCCTATG
M1038	CAAACACTGGCTCACAGATG
M1051	GTAATACGACTCACTATAGGGCCCGGGCCGCCGCCGGCCGCTAACCTTAGCCCCTGCCAGTACGCCGTGGAACGGCCGGTGGGCAGTGGAGAGGGCAGAG
M1052	CATGAATTCAAGCCAGTCTAGGCGACCCCTGTCCGGAGAGGCTGTGAATTACTGCCCCGCCCTCGGGGATGGATCCACAGACCTGCGGCTCACTTGTACAGCTCGTCCATGCC
M1053	GTAATACGACTCACTATAGGGCAAGAACCAGATGATGCTGGGCAGGAGCAGCCCCTCGAAGAACTATGTGATGGCCGTCTCCAGCGGCAGTGGAGAGGGCAGAG
M1054	CATGAATTCTATACGGTGGCCTGTTGTCAGGGCAGCATGAGAACAGTAGAGTGCCAGGGTCGGGTGGCTCCTATCTACTTGTACAGCTCGTCCATGCC
M1055	TAATACGACTCACTATAGGGAGACAGCCGGGCTTCGCGCGTTTTAGAGCTAGAAATAGCAAG
M272	CAGGAAACAGCTATGACC
M389	TCGCCACCATGGTGAGCAAGGGCGAG
M390	CTCTAGACTTTACTTGTACAGCTCGTCCAT
M463	TCCTTCTGTCCAGTGCACCAT
M939	AAAAAAAGCACCGACTCGG
M943	AGGATCTGTGTTCAACCCATT
M944	ACAAAGAAAACCAAGCGTGAC
M947	CTTGAGAAAGGCCACAGTTTC
M948	ACGAACCTCTTCATCTGCTGT
M992	CCTGGACAGCCTGTTGGG
M993	TTCAGTCTGGTGGTGAGACAG
M999	ATGGGTGTTGACCCATTGTT
PP226	CAAGCCAGTCTAGGCGACCC
PP227	CATGAATTCTATACGGTGGCC
Mm HPRT F15	AGGTTTCGAGCCCTGATATTCG
Mm HPRT R15	ATGTGGCAAGGTCAAAAACAGT
Tyr-F	TCTCTGATGGCCATTTTCCTC
Tyr-R	AACATGGGTGTTGACCCATT
Kit-F	GAGGGAAATGGTTTAGTTTGGG
Kit-R	GGGTTTCTGGAGGAGAAAGG
Cdkn1a-fw	CCTGAAGACTGTGATGGGGTA
Cdkn1a-rv	TCTCCGTGACGAAGTCAAAGT
Cdkn2a-fw	GCCGTGATCCCTCTACTTTTT
Cdkn2a-rv	TATCGCACGATGTCTTGATGT
Sry-F2	AAGCGACCCATGAATGCATTCATGGTGTGGT
Sry-R2	GAGGTCGATACTTATAGTTCGGGTATTTCTCTCTGTG

* Regions of homology are underlined.
